# Integrated mRNA-seq and miRNA-seq analysis of goat fibroblasts response to *Brucella Melitensis* strain M5-90

**DOI:** 10.7717/peerj.11679

**Published:** 2021-06-29

**Authors:** Baobao Li, Si Chen, Chengqiang Wang, Qiaoling Chen, Churiga Man, Qi An, Zhenxing Zhang, Zhiyong Liu, Li Du, Fengyang Wang

**Affiliations:** Hainan Key Lab of Tropical Animal Reproduction, Breeding and Epidemic Disease Research, Animal Genetic Engineering Key Lab of Haikou, College of Animal Science and Technology, Hainan University, Hainan Key Lab of Tropical Animal Reproduction, Haikou, Hainan, China

**Keywords:** *Brucella melitensis* strain M5-90, Goat fibroblasts, Transcriptomics, Innate immunity, Adaptive immunity

## Abstract

Brucellosis is a globally zoonotic bacterial disease of humans and various animals including goats, sheep, and cattle. *Brucella melitensis* M5-90, a live attenuated vaccine strain, has been widely used to prevent brucellosis in goats and sheep. However, the molecular mechanisms governing protective immunity response in non-professional phagocytes infected with *B. melitensis* M5-90 have not been fully investigated, especially in goats. In our research, goat fibroblasts were used as in vitro models to determine these mechanisms by transcriptome analysis. After incubating with *B. melitensis* M5-90 3 h, the infected goat fibroblasts were collected at 0 h, 4 h, 24 h, 48 h and 72 h for RNA-seq. The results indicated that there were totally 11,819 differentially expressed genes (DEGs) and 777 differentially expressed (DE) miRNAs found in experiment groups compared with the control groups (|log2(Foldchange)|≥1, FDR<0.05). GO and KEGG enrichment analyses revealed that down-regulated genes were involved in the riboflavin metabolism and positive regulation of IL-8 secretion pathway. The up-regulated genes were mainly involved in adaptive immunity, including TNF signaling pathway, MAPK signaling pathway and JAK/STAT pathway. Additionally, cytokine-cytokine receptor interaction, natural killer cell mediated cytotoxicity and toll-like receptor signaling pathway, which associated with innate immunity pathways, were also induced. Based on the Pearson correlation coefficients and prediction results of TargetScan and miRanda, the miRNA-mRNA networks of *NFKB1*, *IFNAR2* and *IL10RB* were constructed and verified in goat fibroblasts by qPCR, which demonstrated that goat fibroblasts displayed immunomodulatory properties. Our findings provide a deeper insight into the host miRNA-driven *B. melitensis* defense mechanism and reveal the transcriptome changes involved in the innate and adaptive immune response of the goats to *B. melitensis* infection.

## Introduction

Brucellosis, a disease caused by *Brucella* genus members, is a worldwide zoonosis responsible for chronic infections in animals (Pappas et al., 2006). The primary host range for *Brucella* genus is broad, encompassing several animals from which animal products are derived (Whatmore et al., 2014). The infection in farmed animals usually manifests itself as spontaneous abortions and infertility. The *Brucella* genus has recently expanded from six to 11 species, all of which are associated with mammals (Soler-Llorens et al., 2016; Suarez-Esquivel et al., 2020). Among all *Brucella* species, *B*. *melitensis* is the most commonly seen pathogen in goats and has the highest zoonotic potential-causing the most severe cases of human brucellosis (Blasco & Molina-Flores, 2011; Chain et al., 2005). Human brucellosis primarily occurs following mucosal exposure to contaminated aerosols or ingestion of contaminated animal products (Alsaif et al., 2018). Malta fever, chronic debilitating disease, as well as spontaneous abortions and sterility are the main clinical symptoms (Franco et al., 2007). Many countries are now seeing the reemergence of human brucellosis, with estimates of 500,000 individuals each year contracting brucellosis from meat, milk, hair, wool or the amniotic fluids of infected domestic animals (Lacey et al., 2018). The lack of adequate disease control measures in the small ruminant production industry is the main reason for the high prevalence of *B. melitensis* infections (Moreno, 2014).

*B. melitensis* M5-90 vaccine strain, which originated from a virulent *B. melitensis* M28 strain, was generated by acriflavine treatment and continuous passage in cultured chicken embryo fibroblasts (Wang et al., 2011a). Research on the attenuation mechanism of *B. melitensis* M5-90 demonstrated that the single nucleotide polymorphisms, insertion and deletion of those virulence genes, such as *manB*, *tuf2*, *vjbR* and *wboA* gene, contributed to the reduced pathogenicity of the vaccine strain (Li et al., 2018, 2015; Wang et al., 2013; Zhang et al., 2017). At present, the immunization techniques including subcutaneous injection and mucosal immunization are shown to be effective at preventing brucellosis in goats and sheep (Wang et al., 2013). The large-scale application has been limited by safety concerns, especially due to interference with diagnostic tests, pathogenicity for humans and potential to cause abortion in pregnant animals. *B. melitensis* M5-90 has been found to survive and replicate in professional phagocytes (such as macrophages) for long periods of time by evading host immunity to re-emerge later on and systemically disseminate throughout the whole body (Xu et al., 2021). RNA-sequencing analysis of mouse peritoneal macrophage responses to *B. melitensis* M28 and M5-90 infection indicated that the attenuated strain M5-90 had a reduced ability to avoid phagosome-lysosome fusion and activated MAPK pathways as the key regulatory pathway (Wang et al., 2011b). Despite the importance of macrophages in the initial *B. melitensis* M5-90 pathogenesis, a detailed molecular response of non-professional phagocytes infected with the intracellular pathogen has not been fully investigated, especially in goats themselves.

Fibroblasts are the most abundant cell type in the skin or the mucous membrane and play a critical role as a protective barrier against various pathogenic microorganisms. Fibroblasts are able to secrete and respond to cytokines, chemokines, and growth factors (Van Linthout, Miteva & Tschöpe, 2014). Research on mice observed that the type of host cells infected by *B. melitensis* 16M is strongly dependent on the route of infection. The infected cells are mainly macrophages in the intraperitoneal infection and intranasal infection models, while fibroblasts and neutrophils are also infected in the intradermal infection model (Demars et al., 2019). Due to the need to better understand the function of goat fibroblasts in innate and adaptive immune responses, we used high-throughput RNA sequencing (RNA-seq) technology to reveal the dynamic changes from 4 h to 72 h in *B. melitensis* M5-90 infected fibroblasts. The goal of our research is to provide clues on the identification of host candidate genes in immune responses and protective immunity process.

## Materials & methods

### Cell culture

Goat fibroblasts were obtained from the cell bank of the Chinese Academy of Science (Kunming, China) and grown in DMEM (Hyclone, GE, USA) with 10% fetal bovine serum (FBS) (Gibco, Invitrogen, Waltham, MA, USA) in 5% CO_2_ and at 37 °C with 100% humidity. After plating the cells at a density of 5 × 10^5^ cells per well in 6-well plates, they were challenged with 5 × 10^7^ colony-forming units (CFUs) of *B. melitensis* M5-90 in sterile PBS. The above optimal multiplicity of infection (MOIs) was 100 based on the research of fibroblast-like synoviocytes (Scian et al., 2011). To establish a suitable incubation time, the infected goat fibroblasts were washed with PBS three times and then cultured in DMEM with 10% FBS and 30 ug/mL gentamicin at 2 h (h), 3 h, 4 h and 5 h after infection. The cell morphologies were observed by inverted fluorescence microscopy (IX71; OLYMPUS, Tokyo, Japan). The experiments were repeated three times.

### *B. melitensis* M5-90 Infection

*B. melitensis* M5-90 were obtained from the Center of Chinese Disease Prevention and Control (Beijing, China). The strain was incubated on BBL™ Brucella Agar (BD, USA) for 72 h at 37 °C and identified by gram staining. The *B. melitensis* 16S rRNA gene was PCR-amplified, and the resulting sequence was subjected to the BLASTn analysis against the GenBank database (accession No. CP001851.1). Primer sequences were as follow: 16s rRNA-forward 5′-AGAGTTTGATCCTGGCTCAG-3′ and 16s rRNA-reverse 5′-TACGGCTACCTTGTTACGACTT-3′. A standard linear equation for the concentration of *B. melitensis* M5-90 vs absorbance was established using the continuous gradient dilution method. The *B. melitensis* M5-90 strain was frozen at −80 °C with 75% (v/v) glycerol. All the challenge experiments were conducted in a biosafety laboratory.

### RNA extraction and quality assessment

After incubating with *B. melitensis* M5-90 3 h, the infected goat fibroblasts were washed with PBS three times and then cultured in DMEM with 10% FBS and 30 ug/mL gentamicin to eradicate the extracellular *B. melitensis* M5-90. The infected goat fibroblasts were collected at 0 h, 4 h, 24 h, 48 h and 72 h. RNA extraction was performed using the standard TRIzol (15596026; Invitrogen, Waltham, MA, USA) reagent protocol, and DNase I was used to remove contaminating gDNA. The RNA integrity was determined from the electropherogram generated by the Agilent BioAnalyzer. The mRNA library was prepared as follows. Total mRNA was enriched using oligo (dT) beads (Epicenter, Madison, USA) and then ligated to adaptors using T4 RNA ligase II. The mRNA was reverse-transcribed and purified using AMPure XP beads, and the molar concentration was determined for each cDNA library. The Illumina HiSeq™4000 platform was used for sequencing. The raw reads have been deposited to the NCBI Sequence Read Archive (SRA) under accession PRJNA659620. The miRNA library was made using the same protocol as that of the mRNA library except that the miRNA reverse transcription products were purified using 6% polyacrylamide Tris-borate-EDTA, from which 140–160 bp PCR fragments were isolated. The Illumina HiSeq™2500 was used for sequencing. The raw reads have been deposited to the NCBI Sequence Read Archive (SRA) under accession PRJNA659659.

### mRNA sequencing and data analysis

The sequencing data were validated by a series of filtration steps as follows: adapter sequences were discarded; reads containing more than 5% of unknown nucleotides (N) were discarded; and low quality reads were removed. The valid data were mapped to the reference genome (GCF_001704415.1) using HISAT2 (Version 2.0.4), and they were used to assemble transcripts with the reference annotation by StringTie (Version 1.3.4d). Gene expression levels were normalized by fragments per kilobase of exon model per million mapped reads (FPKM). The FPKM calculation equation we used was FPKM=cDNA fragments/Mapped Reads (Million) × Transcript Length (kb). Principal components analysis (PCA) and spearman correlation were performed using the R package (Version 3.6.1). Compared with the control (Ctrl), genes with the |log2 (Fold change) |≥1 and false discovery rate (FDR) <0.05 were considered as differentially expressed genes (DEGs) across the experimental groups by R package edgeR. The expression data for the control (Ctrl), T4h, T24h, T48h and T72h samples were normalized to that of the Ctrl, after which log2 (T4h/Ctrl), log2 (T24h/Ctrl), log2 (T48h/Ctrl) and log2 (T72h/Ctrl) were clustered by Short Time-series Expression Miner (STEM) software (Ernst & Bar-Joseph, 2006). The DEGs of profile 1 and profile 9 that involved in immunity and inflammation processes were selected for further analyzing. The Kyoto Encyclopedia of Genes and Genomes (KEGG) database and Gene Ontology (GO) databases were used for pathway annotation of the DEGs.

### miRNA sequencing and data analysis

Raw reads were subjected to the ACGT101-miR program (LC Sciences, Houston, TX, USA) to remove the low quality raw reads. The criteria for filtration were: (i) adapter sequences were excluded; (ii) < 80% A, G, T or G signal bases were selected; (iii) all four bases (A, T, C, G) were included; (iv) < 2 N bases (ambiguous bases) were included; (v) the small RNA lengths were between 18 and 26 N; (vi) and other RNAs were removed via BLAST against the RFam and Repbase databases. Subsequently, unique sequences with length in 18–26 nucleotide were mapped to specific species precursors in miRBase 22.0 by BLAST search to identify known miRNAs and novel miRNAs. Differentially expressed (DE) miRNAs based on normalized deep-sequencing counts were analyzed by ANOVA and identified by |log2 (Fold change)| ≥1 and FDR<0.05. In order to achieve the integrated analysis of RNA-seq and miRNA-seq, all the DE miRNAs were analyzed by STEM analysis. After the STEM analysis, the clustered profiles 1 (down) and profile 9 (up) were further analyzed. The KEGG database and GO databases were used for pathway annotation of the DE miRNAs.

### MiRNA-mRNA network construction

In order to test the association between paired miRNA-mRNA in the same sample, the Pearson correlation coefficients and FDR values were computed by R package. Generally, it is considered that |P|>0.6 and FDR<0.05 represented interdependency. The biological functions of the negatively correlated DE miRNA-mRNA pairs were analyzed using GO and KEGG pathway enrichment. Furthermore, TargetScan and Miranda were used to predict the target genes of DE miRNAs. The DE miRNAs with values of less than 50 in context percentile score, and those whose maximum energy did not reach −10, were discarded. After applying the above filters, the residual miRNAs were used to construct miRNA-mRNA networks using Cytoscape 3.8.2 software.

### Real-time quantitative PCR (qPCR) validation

We used qPCR to verify the reliability of the RNA sequencing and miRNA sequencing. Total RNA was reverse transcribed according to the manufacturer’s protocol from the M-MLV G III Frist-Strand Synthesis Kit (EB15012; Yugong Biolabs, Lianyungang, China). The qPCR was performed using RealUniversal PreMix (FP201; TIANGEN, Beijing, China) on an ABI 7500 Real-Time PCR System (Applied Biosystems, Foster City, CA, USA). Relative transcriptional levels were determined by the 2^−∆∆Ct^ method (Livak & Schmittgen, 2001). EEF1A1 and β-actin were the reference genes. For miRNA validation, goat fibroblasts were grown in 6-well plates and transfected with 100 pmol miRNA mimics utilizing 7.5 µL Lipofectamine 3000 transfection reagent (L3000008; Invitrogen, Waltham, MA, USA). All experiment control samples were treated with an equal concentration of a non-targeting control mimics sequence (Cy3-NC). After 24 h, the RNA was extracted using the standard TRIzol (15596026; Invitrogen, Waltham, MA, USA) reagent protocol. The miRcute Plus miRNA First-Strand cDNA Kit (KR211; TIANGEN, Beijing, China) was used to reverse transcribe total RNA from the cells. The qPCR was conducted following the protocol from the miRcute Plus miRNA qPCR Kit (FP411; TIANGEN, Beijing, China). The specific stem-loop reverse primer sequence was 5′-AGTGCAGGGTCCGAGGTA3’. Chi-miR-10b-3p was the reference miRNA. All primers were listed in the Supplemental Files. The experiments were repeated three times.

### Statistical analyses

All the qPCR results are expressed as mean ± SEM. *T*-test was performed on the qPCR data to determine whether the difference is statistically significant (Schmittgen & Livak, 2008). *p* < 0.05 were considered to be statistically significant.

## Results

### The quality of cDNA libraries and sequencing data

**Figure 1:**
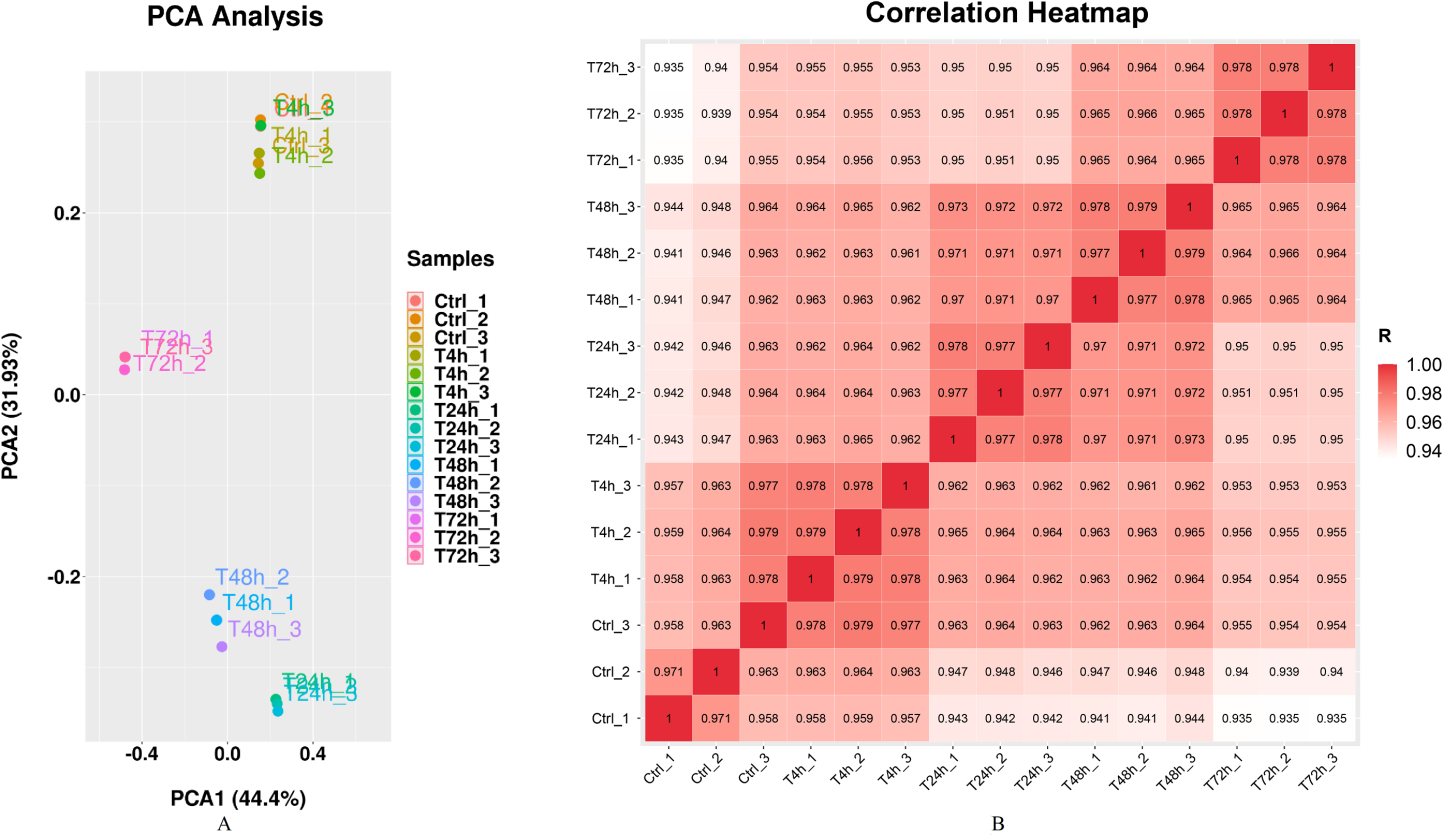
Principal component analysis (PCA) plot and correlation heatmap of samples. (A) PCA score plot. Different colors denote the investigated stages. (B) Correlation heatmap of samples. The gradient color barcode at the right indicates the minimum value in white and the maximum in red. If one sample is highly similar to another one, the correlation value between them is very close to 1.

Gram staining and Kirzovsky staining were consistent with the standard staining results. The PCR-amplified 16s rRNA fragment was 1,465 bp length, and shared 99.06% homology with that in the GenBank database. The standard linear equation was: Y = 10^10^ X + 4 × 10^6^, in which X represented the OD value, and Y represented the CFU/mL. The optimal incubation time was 3 h. After infection with *B. melitensis* M5-90 (MOI = 100), the morphologies of goat fibroblasts were recorded at 4 h, 24 h, 48 h and 72 h. Significant cell pathological changes, which included cell necrosis and cell drift decreases, were observed at 72 h. The RNA integrity number (RIN) values of all samples were consistently ≥ 9.6, which were generally considered high quality. Q30 scores were above 94.20% (Supplemental File). The results of mRNA sequence alignment analysis were summarized in Table 1. Mapped ratio was between 51.47% and 59.58%. The incomplete RefSeq database and species divergence may lead to the low uniquely mapped reads in our research. The Principal Components Analysis (PCA) showed that the 1st principal component (PC1) contained 44.4% of the variance, while the 2nd principal component contained 31.93% of the variance. The clustering data points were clearly visible among groups (Fig. 1A). The correlation heatmap displayed the good agreement among each group (Fig. 1B).

**Table 1:** mRNA sequence alignment analysis.

Sample	Valid reads	Mapped reads	Unique Mapped reads	Multi Mapped reads	PE Mapped reads	Reads map to sense strand	Reads map to antisense strand	Non-splice reads	Splice reads
Ctrl_1	51,407,804	27,524,140 (53.54%)	13,364,040 (26.00%)	14,160,100 (27.54%)	20,469,800 (39.82%)	13,093,012 (25.47%)	13,080,483 (25.44%)	10,537,410 (20.50%)	15,636,085 (30.42%)
Ctrl_2	42,122,724	22,862,663 (54.28%)	10,900,499 (25.88%)	11,962,164 (28.40%)	16,889,304 (40.10%)	10,856,772 (25.77%)	10,857,246 (25.78%)	8,760,276 (20.80%)	12,953,742 (30.75%)
Ctrl_3	51,381,438	26,599,181 (51.77%)	13,104,485 (25.50%)	13,494,696 (26.26%)	19,231,204 (37.43%)	12,610,872 (24.54%)	12,637,511 (24.60%)	9,940,042 (19.35%)	15,308,341 (29.79%)
T4h_1	47,879,826	24,644,183 (51.47%)	12,137,863 (25.35%)	12,506,320 (26.12%)	17,736,424 (37.04%)	11,708,376 (24.45%)	11,722,489 (24.48%)	9,287,250 (19.40%)	14,143,615 (29.54%)
T4h_2	55,128,232	28,735,638 (52.13%)	14,111,096 (25.60%)	14,624,542 (26.53%)	20,897,678 (37.91%)	13,631,578 (24.73%)	13,645,623 (24.75%)	10,713,933 (19.43%)	16,563,268 (30.04%)
T4h_3	41,173,302	22,456,730 (54.54%)	10,493,022 (25.49%)	11,963,708 (29.06%)	16,682,552 (40.52%)	10,650,872 (25.87%)	10,627,927 (25.81%)	8,412,818 (20.43%)	12,865,981 (31.25%)
T24h_1	41,943,922	24,276,721 (57.88%)	11,007,180 (26.24%)	13,269,541 (31.64%)	18,698,622 (44.58%)	11,309,913 (26.96%)	11,290,045 (26.92%)	8,931,152 (21.29%)	13,668,806 (32.59%)
T24h_2	44,351,258	25,269,329 (56.98%)	12,091,705 (27.26%)	13,177,624 (29.71%)	19,130,624 (43.13%)	12,007,658 (27.07%)	12,008,790 (27.08%)	9,043,462 (20.39%)	14,972,986 (33.76%)
T24h_3	39,233,130	22,219,950 (56.64%)	10,515,744 (26.80%)	11,704,206 (29.83%)	16,796,354 (42.81%)	10,515,600 (26.80%)	10,506,614 (26.78%)	8,060,704 (20.55%)	12,961,510 (33.04%)
T48h_1	43,168,614	24,588,359 (56.96%)	11,305,718 (26.19%)	13,282,641 (30.77%)	18,606,606 (43.10%)	11,485,466 (26.61%)	11,475,805 (26.58%)	9,010,338 (20.87%)	13,950,933 (32.32%)
T48h_2	53,373,502	31,801,679 (59.58%)	12,073,054 (22.62%)	19,728,625 (36.96%)	24,936,620 (46.72%)	13,333,407 (24.98%)	13,327,207 (24.97%)	12,431,263 (23.29%)	14,229,351 (26.66%)
T48h_3	49,697,284	27,164,537 (54.66%)	13,182,590 (26.53%)	13,981,947 (28.13%)	20,022,614 (40.29%)	12,811,863 (25.78%)	12,844,434 (25.85%)	9,894,482 (19.91%)	15,761,815 (31.72%)
T72h_1	47,659,452	25,476,944 (53.46%)	11,374,681 (23.87%)	14,102,263 (29.59%)	18,782,232 (39.41%)	11,549,642 (24.23%)	11,517,871 (24.17%)	9,910,845 (20.80%)	13,156,668 (27.61%)
T72h_2	51,005,958	26,916,478 (52.77%)	12,643,191 (24.79%)	14,273,287 (27.98%)	19,597,704 (38.42%)	1,249,721 0(24.50%)	12,451,142 (24.41%)	10,208,633 (20.01%)	14,739,719 (28.90%)
T72h_3	48,795,348	25,951,301 (53.18%)	12,130,318 (24.86%)	13,820,983 (28.32%)	18,873,720 (38.68%)	12,019,714 (24.63%)	11,980,782 (24.55%)	9,720,530 (19.92%)	14,279,966 (29.27%)

### DEGs and DE miRNAs among groups

**Figure 2:**
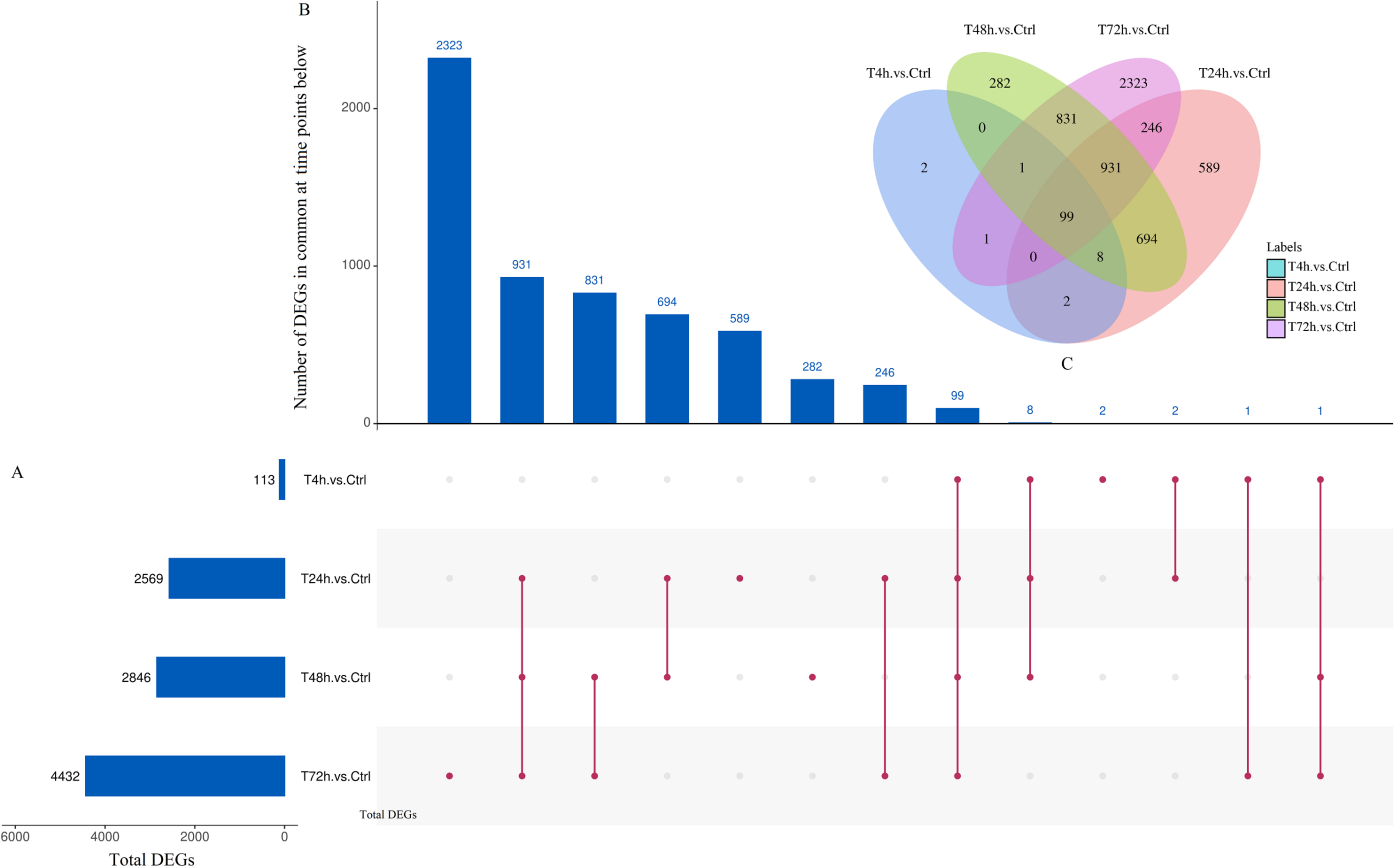
UpSet plot and Venn diagram of differentially expressed genes (DEGs). **(**A) Graph of total number of DEGs (*X* axis) at each time point (*Y* axis). (B) Intersection of sets of genes at multiple time points. Each column corresponds to a set of time points (dots connected by lines below the *X* axis) containing the same DEGs. The number of genes in each set appears above the column. (C) ****The Venn diagram. The number of DEGs among comparisons of T4h vs. Ctrl, T24h vs. Ctrl, T48h vs. Ctrl and T72h vs. Ctrl.

**Figure 3:**
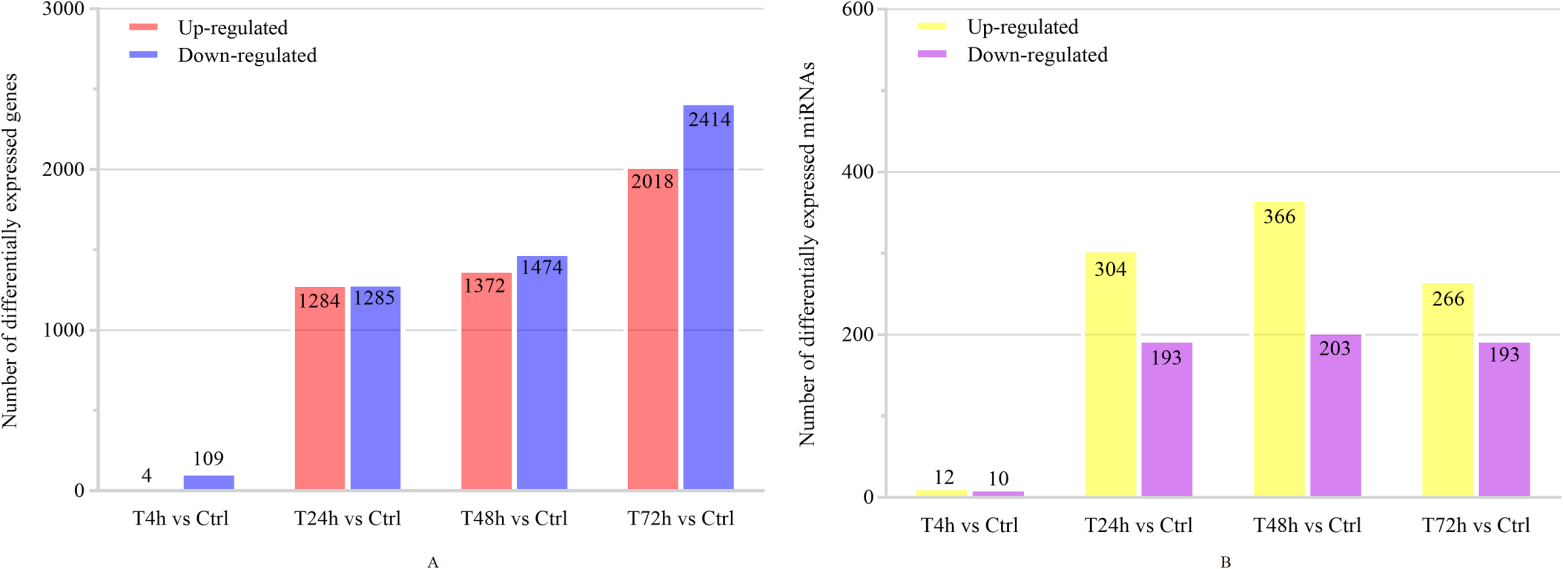
The numbers of DEGs and differentially expressed (DE) miRNAs in *B. melitensis* M5-90 infected cells. (A) The number of up-regulated and down-regulated DEGs at different time points (FDR<0.05). (B) The number of up-regulated and down-regulated DE miRNAs at different time points (FDR<0.05).

**Figure 4:**
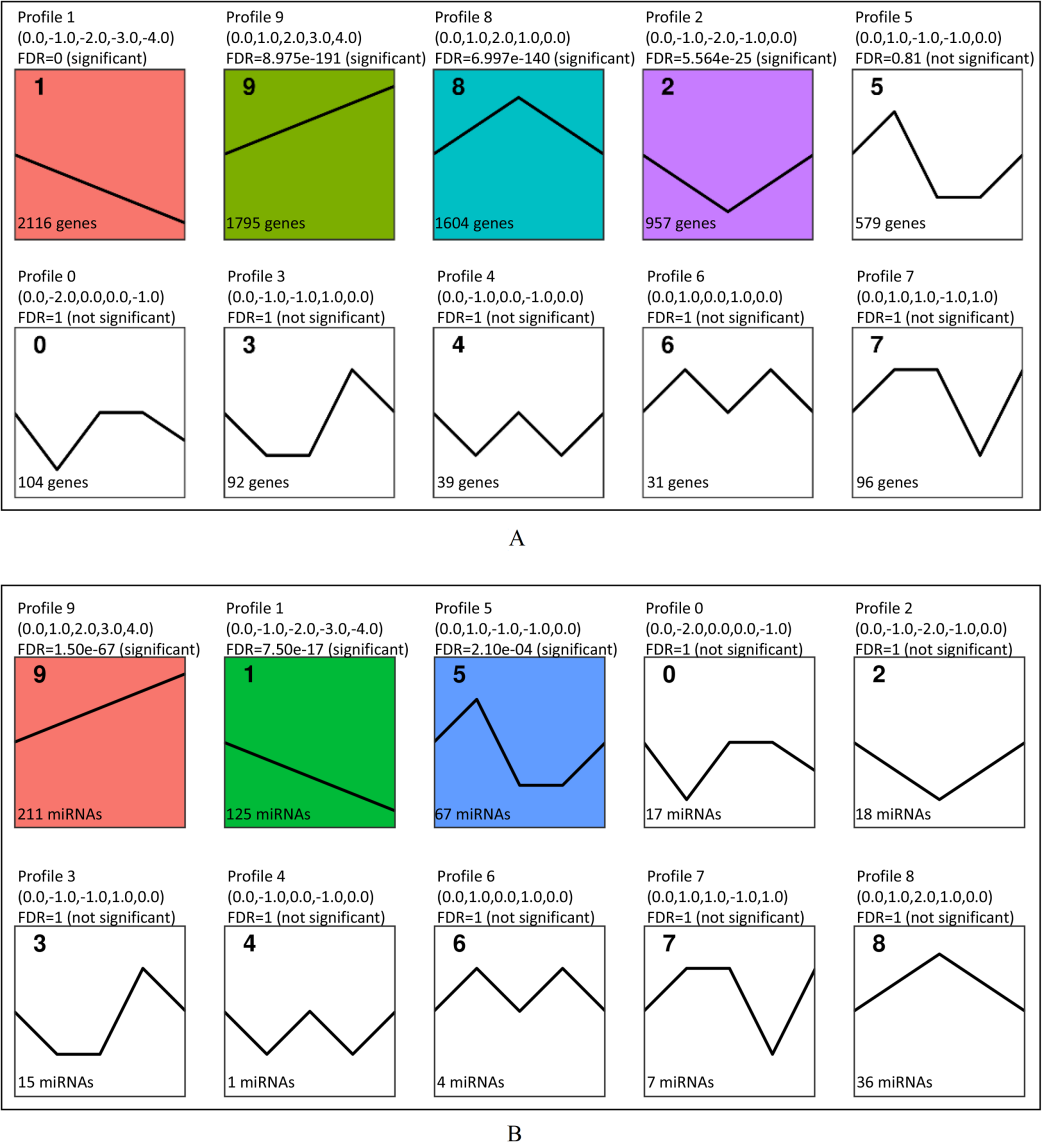
Short Time-series Expression Miner (STEM) analysis of DEGs and DE miRNAs. (A) STEM analysis of 7,413 DEGs among all time points. The colored profiles were with significance (FDR<0.05). (B) STEM analysis of 501 DE miRNAs. The colored profiles were with significance (FDR<0.05).

Based on the thresholds of *P* < 0.05, the differential expression analysis returned 12,441 DEGs and 2,547 DE miRNAs (unpublished data). In order to overcome the false positive problem, the *P* values were adjusted with the Benjamini and Hochberg method to control the false discovery rate. We used FDR instead of *P* values and set the FDR threshold of 0.05. A total of 11,819 DEGs and 777 DE miRNAs were screened across all time points (FDR<0.05). The UpSet diagram clearly showed that 99 mRNAs were consistently expressed throughout the entire duration of the experiment (Fig. 2). As shown in Fig. 3A, 4, 1,284, 1,372 and 2,018 DEGs were up-regulated among the comparisons of T4h vs Ctrl, T24h vs Ctrl, T48h vs Ctrl and T72h vs Ctrl, respectively, whereas 109, 1,285, 1,474 and 2,414 DE mRNAs were down-regulated, respectively. Moreover, the differential expression analysis on the miRNAs revealed that the number of up-regulated miRNAs far exceeded that of the down-regulated miRNAs between the different groups (Fig. 3B).

### Short time-series expression miner (STEM) analysis

The results of STEM analysis on the screened 7,413 DEGs are shown in Fig. 4. Four significant profiles were clustered, including profile 1, profile 9, profile 8 and profile 2. The expression tendency of 2,116 DEGs in profile 1 showed a downwards trend and 1,795 DEGs in profile 9 showed an upwards trend during B. *melitensis* M5-90 infection at 0–72 h (Fig. 4A). Among them, 413 up-regulated genes were associated with MAPK signaling pathway, cytokine-cytokine receptor interaction, TNF signaling pathway, toll-like receptor signaling pathway, Jak-STAT signaling pathway, NOD-like receptor signaling pathway and RIG-I-like receptor signaling pathway (Table 2). The STEM analysis showed that, among the 501 DE miRNAs that were screened, three profiles were significant (profiles 9, 1 and 5). The expression tendency of each miRNA in profile 9 showed an upward trend, whereas the miRNA in profile 1 showed a downward tendency (Fig. 4B).

**Table 2:** The number of DEGs associated with different KEGG pathways in STEM profile 1 and profile 9.

Pathway ID	Pathways	No. of DEGs In profile 1 (Down)	*P* value	No. of DEGs In profile 9 (Up)	*P* value
ko04010	MAPK signaling pathway	44	0.999982	112	1.81E−09
ko04060	Cytokine-cytokine receptor interaction	14	0.965792	41	3.05E−07
ko04668	TNF signaling pathway	10	0.969333	31	1.29E−05
ko04620	Toll-like receptor signaling pathway	8	0.972678	21	0.007392
ko04630	Jak-STAT signaling pathway	13	0.939661	26	0.010389
ko04621	NOD-like receptor signaling pathway	9	0.82601	17	0.020949
ko04622	RIG-I-like receptor signaling pathway	3	0.997776	14	0.038433
ko04064	NF-kappa B signaling pathway	2	0.999968	15	0.10447
ko04660	T cell receptor signaling pathway	6	0.99242	15	0.161475
ko04662	B cell receptor signaling pathway	5	0.984932	12	0.211995
ko04514	Cell adhesion molecules (CAMs)	25	0.238905	23	0.215574
ko04664	Fc epsilon RI signaling pathway	9	0.32683	9	0.219989
ko04370	VEGF signaling pathway	10	0.645487	12	0.246406
ko04062	Chemokine signaling pathway	11	0.993438	19	0.422539
ko04512	ECM-receptor interaction	12	0.949205	16	0.511486
ko04666	Fc gamma R-mediated phagocytosis	12	0.878155	13	0.663354
ko04350	TGF-beta signaling pathway	23	0.310551	17	0.683841
	In total	216		413	

### GO and KEGG pathway analyses

**Figure 5:**
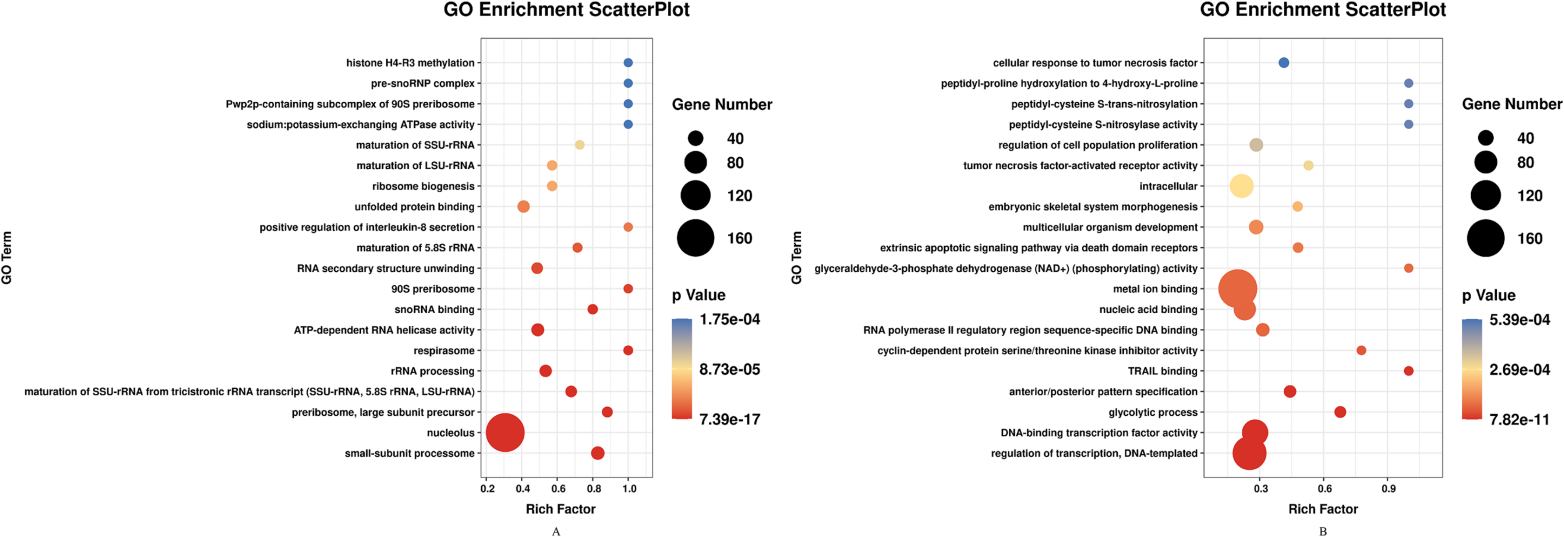
GO enrichment analysis of DEGs in STEM profile 1 (Down) and profile 9 (Up) (Top 20, FDR<0.05). (A) GO enrichment analysis of DEGs in STEM profile 1 (Down). (B) GO enrichment analysis of DEGs in STEM profile 9 (Up). The DEGs common to all of T4h vs Ctrl, T24h vs Ctrl, T48h vs Ctrl and T72h vs Ctrl were used for GO enrichment. Rich factor represents the enrichment of DEGs. A higher rich factor corresponds to a shifting of gene set constituents towards either end of the ranked list representing strongly positive or negative correlations.

**Figure 6:**
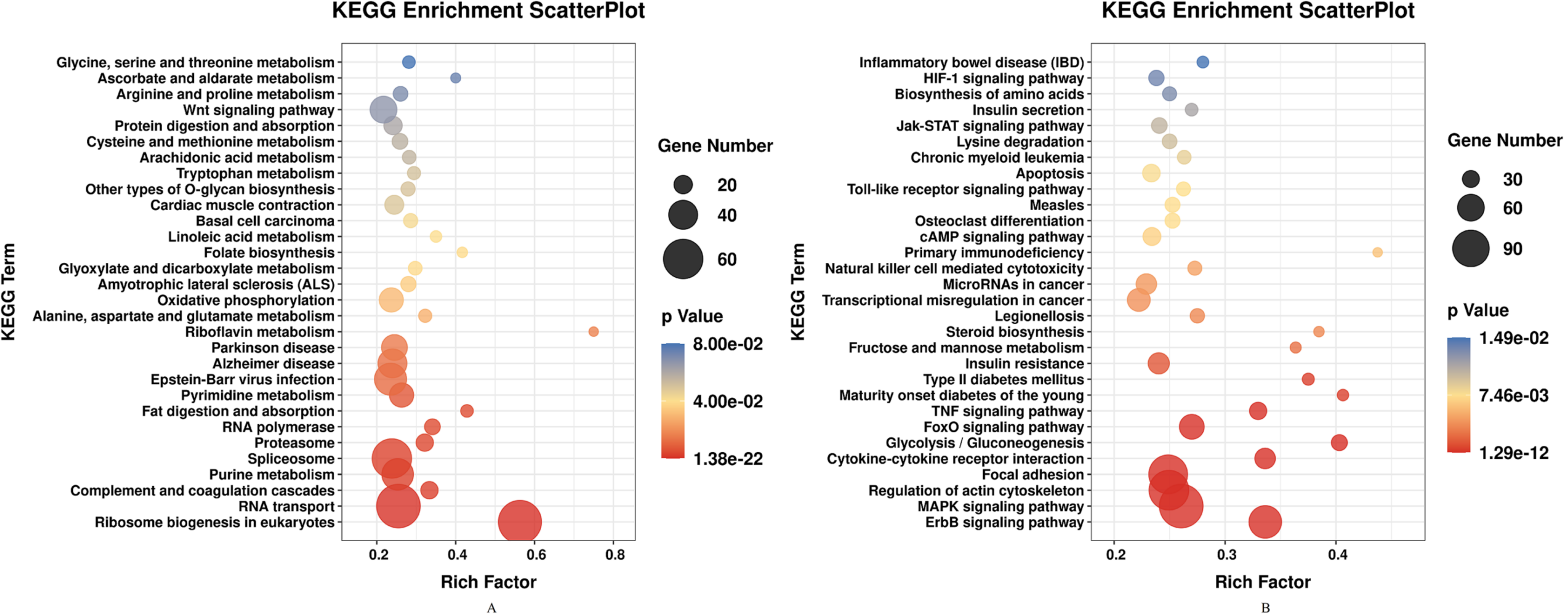
KEGG pathway enrichment analysis of DEGs in STEM profile 1 (Down) and profile 9 (Up) (Top 30, FDR<0.05). (A) KEGG pathway enrichment analysis of DEGs in STEM profile 1 (Down). (B) KEGG pathway enrichment analysis of DEGs in STEM profile 9 (Up). The DEGs common to all of T4h vs Ctrl, T24h vs Ctrl, T48h vs Ctrl and T72h vs Ctrl were used for KEGG pathway enrichment analysis. Rich factor represents the enrichment of DEGs.

DEGs of profile 1 and profile 9 were used for the GO and KEGG pathway analyses. GO analysis indicated that most down-regulated DEGs participated in respirasome, 90S preribosome and positive regulation of interleukin-8 secretion (Fig. 5A). KEGG pathway was predominantly enriched in riboflavin metabolism (Fig. 6A). Conversely, the up-regulated DEGs of profile 9 were enriched in TRAIL binding and glyceraldehyde-3-phosphate dehydrogenase activity GO terms (Fig. 5B). It is worth noting that most statistically significant KEGG pathways were associated with adaptive immunity pathways, including TNF signaling pathway, MAPK signaling pathway and JAK/STAT pathway (Fig. 6B). However, several innate immunity pathways also were induced, for instance, cytokine–cytokine receptor interaction, natural killer cell mediated cytotoxicity and toll-like receptor signaling pathway. Moreover, the GO function and KEGG pathway enrichment analyses indicated that total 11,819 DEGs across all time points were enriched in mitochondrial matrix, poly(A) RNA binding, spliceosome and ribosome biogenesis in eukaryotes (FDR<0.05) (Supplemental File).

### qPCR of DEGs and DE miRNAs

**Figure 7:**
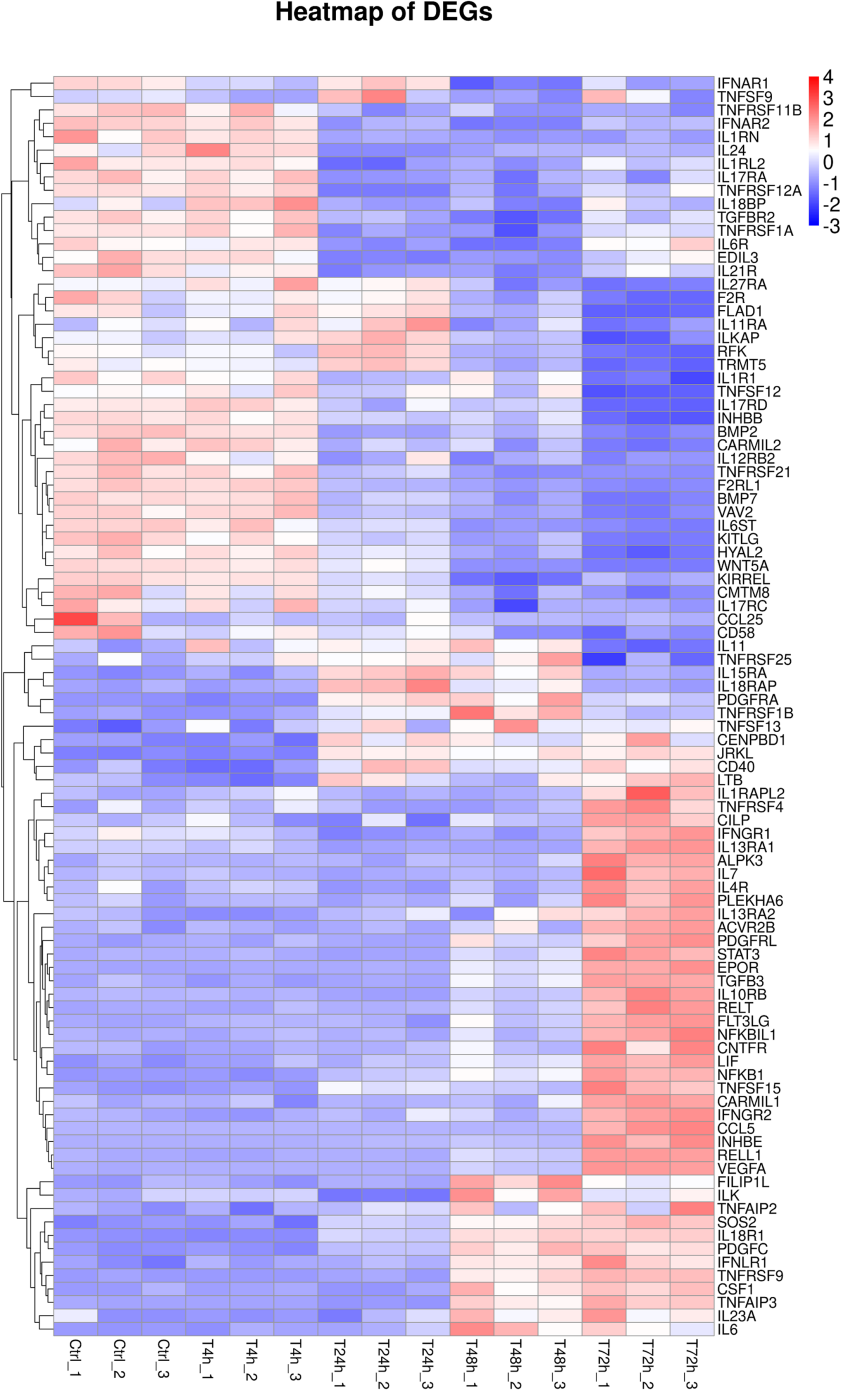
Heatmap of DEGs. During 0 h to 72 h, 94 DEGs clustered in the cytokine–cytokine receptor interaction (ko04060) and TNF signaling pathway (ko04668) were performed.

**Figure 8:**
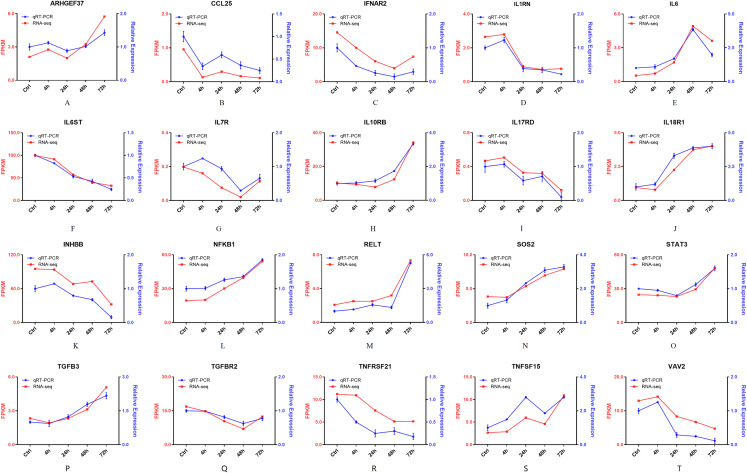
(A–T) The qPCR results of 20 DEGs. Twenty DEGs associated with the cytokine-cytokine receptor interaction and chemokine signaling pathway, were checked by qPCR. Blue lines represent the relative expression of qPCR. Red lines represent the FPKM of RNA-seq.

Furthermore, 94 DEGs clustered in the cytokine-cytokine receptor interaction (ko04060) and TNF signaling pathway (ko04668) were used to create the heat map. The associated DEGs, which were not in profile 1 and profile 9, were excluded. Approximately two thirds of DEGs were gradually increased (Fig. 7). In order to verify the RNA-seq results, the expression of 20 DEGs that associated with the cytokine-cytokine receptor interaction and chemokine signaling pathway, were checked by qPCR. The expression tendency of 20 DEGs were verified as consistent by qPCR and RNA-seq techniques (Fig. 8). The genes in the cytokine-cytokine receptor interaction and chemokine signaling pathway showed an upward tendency, indicating that the host cells released inflammatory molecules after infection.

### miRNA-mRNA network identification

**Figure 9:**
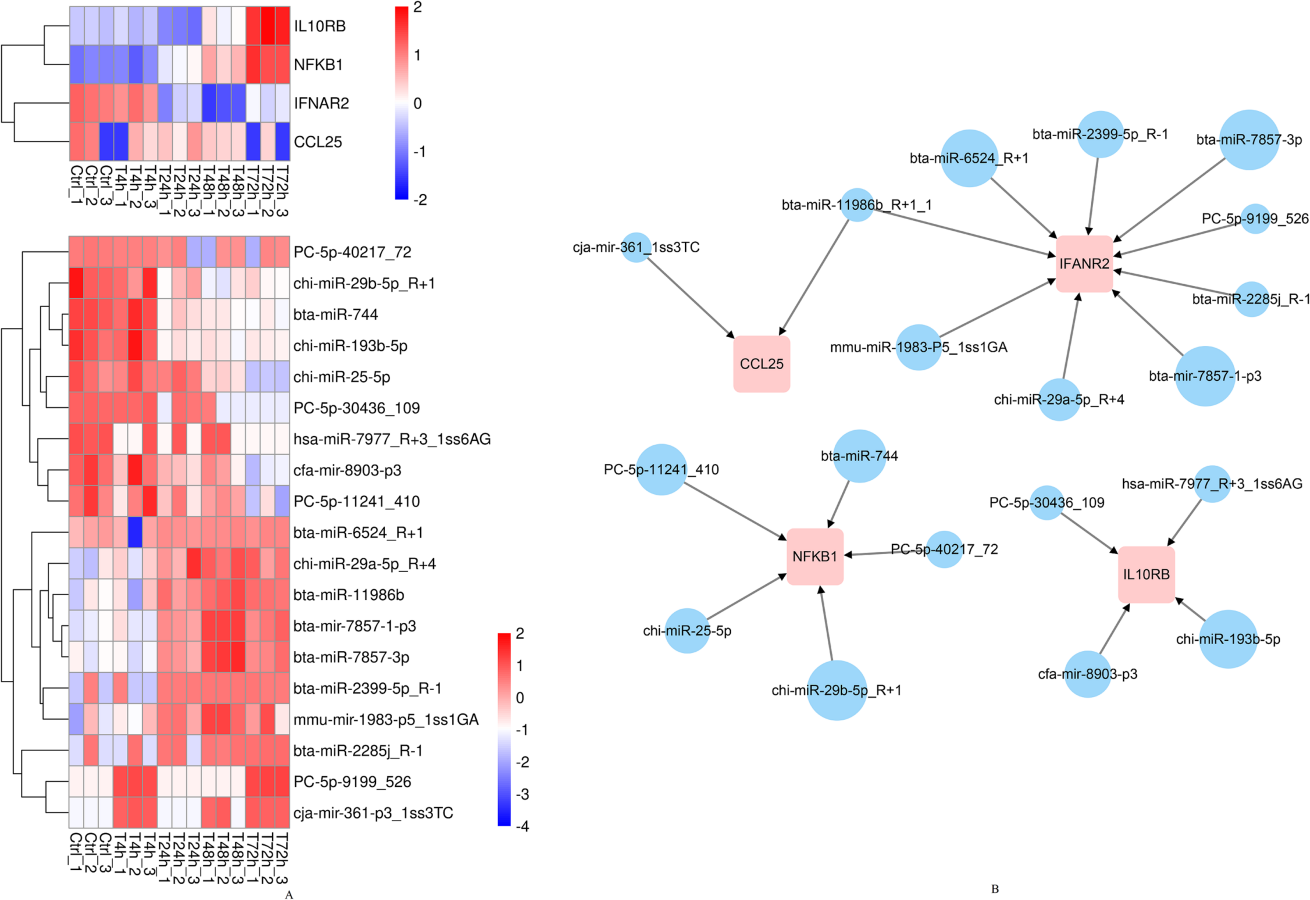
The heatmap and regulatory networks of target DEGs and DE miRNAs. (A) The heatmap of target DEGs and DE miRNAs. (B) The regulatory networks of target DEGs and DE miRNAs. Red box represents DEGs. Blue circle represents DE miRNAs. The size of circles roughly represents the calculated context score percentile by TargetScan 7.0 software.

**Figure 10:**
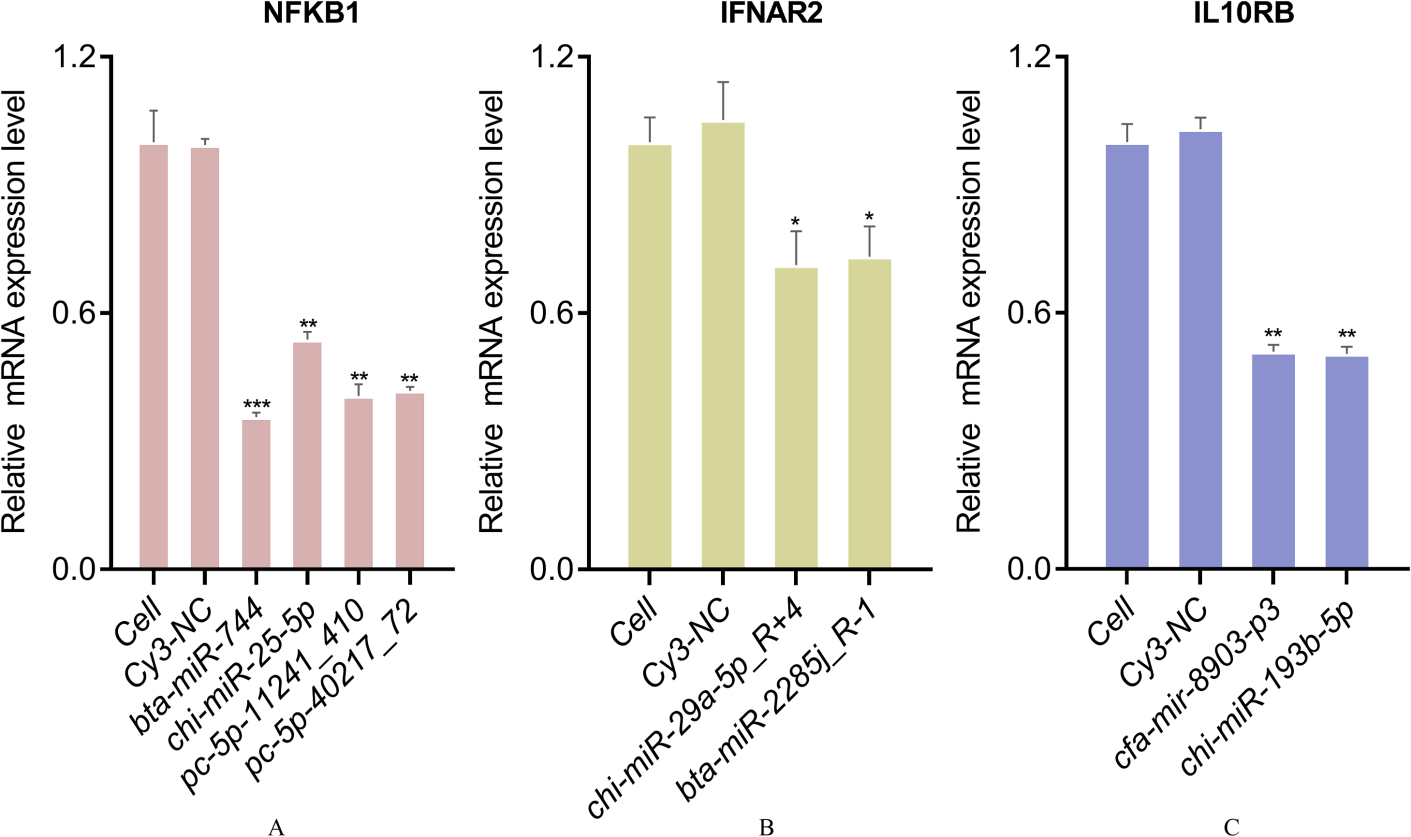
qPCR validation of DGEs in goat fibroblasts transfected with 100 pmol miRNA mimics. (A) qPCR validation of relative expression of *NFKB1* gene. (B) qPCR validation of relative expression of *IFNAR2* gene. (C) qPCR validation of relative expression of *IL10RB* gene. (*, *p* < 0.05; **, *p* < 0.01; ***, *p* < 0.01).

According to the above results, *CCL25*, *IL10RB*, *NFKB1*, and *IFNAR2* were selected for the further miRNA-mRNA network construction. Based on the Pearson correlation coefficients and FDR values, 19 miRNAs were selected. The heat map results revealed that bta-mir-7857-1-p3, bta-miR-7857-3p, bta-miR-11986b, bta-miR-2399-5p_R-1, bta-miR-6524_R+1, chi-miR-29b-5p_R+1, PC-5p-30436_109, mmu-mir-1983-p5_1ss1GA, hsa-miR-7977_R+3_1ss6AG, PC-5p-9199_526, cja-mir-361-p3_1ss3TC and *CCL25* showed poor repeatability within each group (Fig. 9A). Combined with TargetScan and miRanda results, 19 miRNAs were used to build the network to identify crucial miRNAs and genes in the regulatory network (Fig. 9B). Further qPCR results indicated that 10 miRNAs had expression profiles consistent with the miRNA sequencing data (Supplemental File). Finally, eight miRNA mimics were synthesized and transfected into goat fibroblasts (Table 3). The expression of the *NFKB1* gene was dramatically down-regulated with the bta-miR-744 mimic transfected (Fig. 10A), whereas chi-miR-29a-5p_R+4 and bta-miR-2285j_R-1 each decreased the expression level of *IFNAR2* (Fig. 10B). Expression of *IL10RB* gene was found to be down-regulated by cfa-mir-8903-p3 and chi-miR-193b-5p (Fig. 10C).

**Table 3:** Sequence of miRNA mimics.

miRNA	Sense (5′–3′)	Antisense (5′–3′)
bta-miR-744 mimic	UGCGGGGCUAGGGCUAACAGCA	CUGUUAGCCCUAGCCCCGCAUU
chi-miR-25-5p mimic	AGGCGGAGACUUGGGCAAUUGCU	CAAUUGCCCAAGUCUCCGCCUUU
PC-5p-11241_410 mimic	GCGGGCGAGGGUCCGGGGGC	CCCCGGACCCUCGCCCGCUU
PC-5p-40217_72 mimic	AAGGUGACUUUUUAUAUGCCCUCU	AGGGCAUAUAAAAAGUCACCUUUU
chi-miR-29a-5p_R+4 mimic	ACUGAUUUCUUUUGGUGUUCAGAGU	UCUGAACACCAAAAGAAAUCAGUUU
bta-miR-2285j_R-1 mimic	AAAAACCAGAACGAACUUUUU	AAAGUUCGUUCUGGUUUUUUU
cfa-mir-8903-p3 mimic	AUCUGGGGUAGGGCCUGGGAAU	UCCCAGGCCCUACCCCAGAUUU
chi-miR-193b-5p mimic	CGGGGUUUUGAGGGCGAGAUGA	AUCUCGCCCUCAAAACCCCGUU

## Discussion

Previous research focused on identification of *B. melitensis* M5-90 virulence factors (Li et al., 2015; Zhang et al., 2020, 2017) and elucidating unique gene expression profile of mouse infected macrophages (Wang et al., 2011b; Xu et al., 2021). In macrophages, it has already been observed that the two-component system BvrR/BvrS of *B. meliten*sis is required to modulate bacterial physiology and the shift from extracellular to intracellular life (Martín-Martín et al., 2012). The type IV secretion system directs the intracellular trafficking of the *brucella* and modulates host immune responses (Roop et al., 2021). Considering that *Brucella* proliferates within professional and nonprofessional phagocytic host cells (Guzman-Verri et al., 2001), our research is the first to illustrate the immune response occurring in goat fibroblasts during infection with *B. melitensis* M5-90. The above results show that fibroblasts are—in contrast to their traditional view of being solely matrix-producing cells—cells with important immunomodulatory properties, playing a pivotal role in several aspects of the innate and adaptive immune response among other functions.

It is worth noting that most up-regulated DEGs were enriched in adaptive immunity pathways, including TNF signaling pathway, MAPK signaling pathway and JAK/STAT pathway. Considering the remarkable changes of TNF signaling pathway, we initially focused on MAPK signaling pathway and NF-κB signaling pathway. *NFKB1* was significantly up-regulated in goat fibroblasts infected with *B. melitensis* strain M5-90. In mammalian cells, NF-κB consists of five subunits; namely, p65, RelB, c-Rel, p50, and p52. Unlike the other subunits, NFKB1 and NFKB2 proteins are synthesized as long precursors (p105 and p100, respectively), and are proteolytically cleaved to p50 and p52, respectively. Following lipopolysaccharide stimulation in macrophages, p50:p50:Bcl-3 complexes negatively regulate tumor necrosis factor α (TNF-α), interleukin-1a (IL-1a), and interleukin-1b (IL-1b) expression, while activating anti-inflammatory interleukin-10 (IL-10) gene transcription (Wessells et al., 2004). In addition, we noticed a potential relative pathway named “TRAIL binding”. Seven up-regulated DEGs, including LOC108638473, LOC108635488, LOC108635335, LOC108634969, LOC102175959, LOC108635411 and LOC108638455, were enriched in this pathway. These genes encode proteins which interact selectively and non-covalently with TNF-related apoptosis inducing ligand (TRAIL), a member of the TNF ligand family that rapidly induces apoptosis (Pan et al., 1997).

Interestingly, the innate immune responses were also observed in goat fibroblasts after infection. The up-regulated DEGs were prominently enriched in cytokine-cytokine receptor interaction pathway and functioned as a part of the receptor complex, for instance, *IL-10RB*, *IL-12RB2*, *IL-13RA1*, *IL18R1*, *IL6R*, *IFNAR1*, *IFNAR2, IFNGR1* and *IFNGR2*. *IL-10RB* encodes IL-10RB protein, which is a component of heterodimeric IL-10RA (Kotenko, 2002). Coexpression of IL-10RA and IL-10RB is essential for IL10-induced signal transduction, which plays pleiotropic roles in inflammatory and immune responses. *IL-10RB* and three other interferon receptor genes, *IFAR2*, *IFNAR1*, and *IFNGR2*, form a Class II cytokine receptor (CRF2) gene cluster located in a small region (Langer, Cutrone & Kotenko, 2004). *IFNAR1* and *IFNAR2* encoded subunits of the Type I interferon receptor, while *IFNGR1* and *IFNGR2* encoded subunits of the Type II interferon (IFN-γ) receptor. IFN-γ affects diverse aspects of innate immunity and has strong effects on cell-mediated immunity. The IFNγ-producing CD4^+^ T cells are key actors in the process of eliminating *Brucella* (Machelart et al., 2017). MiR-744 is known to be associated with the innate immunity pathway and acts as a tumor suppressor, thereby inhibiting cell proliferation and promoting cell apoptosis (Pilson et al., 2020; Yuan et al., 2020). Meanwhile, the cytokine encoded genes, including *IL-6*, *IL-7*, *IL-11*, *IL-23A* and *IL-24*, were significantly changed in our RNA-seq data, which were partially consistent with previous studies (Avila-Calderón et al., 2020; Zhou et al., 2021). IL-6 is a pleiotropic, pro-inflammatory cytokine produced by a variety of cell types, including lymphocytes, monocytes, and fibroblasts. It not only promotes innate immunity, but also regulates adaptive immunity. Interleukin-8 (IL-8), generally acknowledged as one marked chemokine response to the infection of *Brucella* (including *B. melitensis* H38), was undetected in our original RNA-seq data (Delpino, Fossati & Baldi, 2009). The incomplete reference database may lead to the missing annotation of IL-8. However, GO analysis indicated that the down-regulated DEGs (*WNT5A*, *F2RL1*, *HYAL2*, *F2R*, *CD58* and *LOC100861338)* significantly participated in positive regulation of IL-8 secretion, which may further result in the incapable detecting of *IL-8* expression.

Additionally, one special pathway was found in our model. Three down-regulated DEGs, including *TRMT5*, *RFK* and *FLAD1*, were predominantly associated with riboflavin metabolism KEGG pathways. *RFK* encodes riboflavin kinase, which catalyzes the phosphorylation of riboflavin (vitamin B2) to synthesize two key redox cofactors, flavin mononucleotide (FMN) and adenine dinucleotide (FAD). *FLAD1* encodes the enzyme that catalyzes adenylation of FMN to form FAD coenzyme. Flavin biosynthesis is essential for *B. abortus* intracellular survival and replication inside a macrophagic cell line and mice (Bonomi et al., 2010; Serer et al., 2019). In goat fibroblasts, the down-regulation of riboflavin metabolism may lead to slow down the host oxidation-reduction (redox) reactions.

Previous study identified a Black Angus herd sire which was confirmed to be genetically resistant to in vitro and in vivo challenge of *B. abortus* (Qureshi, Templeton & Adams, 1996). It means that the overall level of genetic resistance to *Brucella* can be increased at population levels by using selective breeding programs. However, the lack of valid candidate genes limits the application of molecular marker-assisted selection (MAS) technology. Our findings provide insight into the host miRNA-driven *B. melitensis* defense mechanism and reveal the transcriptome changes involved in the innate and adaptive immune response of goats to *B. melitensis* infection. These newly discovered host genes and miRNAs-mRNAs networks are potential targets for the construction of genetically edited brucellosis-resistant animal models and will greatly facilitate genomics research in goats. Further understanding of the goat immune response against *B. melitensis* M5-90 now awaits in vivo confirmation.

## Conclusions

Utilizing the RNA sequencing and small RNA sequencing technologies, our research demonstrated transcriptomes changes in *B. melitensis* M5-90 infected goat fibroblasts compared with control cells. A total of 11,819 genes and 777 miRNAs were differentially expressed within 72 h of infection. Most statistically significant KEGG pathways were associated with adaptive immunity pathways, including TNF signaling pathway, MAPK signaling pathway and JAK/STAT pathway. The major significantly enriched pathways in “innate immunity” were cytokine-cytokine receptor interaction, natural killer cell mediated cytotoxicity and toll-like receptor signaling pathway. Moreover, several characteristic pathways in goat fibroblasts, involving positive regulation of IL-8 secretion, riboflavin metabolism, TRAIL binding and glyceraldehyde-3-phosphate dehydrogenase activity were observed. Numerous differentially expression genes and miRNAs related to host protective immunity, including *ARHGEF37*, *CCL25*, *IFNAR2*, *IL10RB*, *IL17RD, IL18R1*, *IL1RN*, *IL6*, *IL6ST*, *IL7R*, *INHBB*, *NFKB1*, *RELT*, *SOS2*, *STAT3*, *TGFB3*, *TGFBR2*, *TNFRSF21*, *TNFSF15*, *VAV2*, *bta-miR-744*, *PC-5p-40217_72*, *PC-5p-11241_410*, *chi-miR-25-5p*, *bta-miR-11986b_R+1_1*, *mmu-miR-1983-P5_1ss1GA*, *chi-miR-29a-5p_R+4*, *bta-miR-2285j_R-1*, *cfa-mir-8903-p3*, *chi-miR-193b-5p*, were confirmed by qPCR. Additionally, the miRNA-mRNA regulated networks of *IL10RB, NFKB1, and IFNAR2* were verified by qPCR, which demonstrated that goat fibroblasts displaying immunomodulatory properties.

## Supplemental Information

10.7717/peerj.11679/supp-1 Supplemental Information 1 Supplementary File. Supplementary File for publication

## Additional Information and Declarations

### Data Availability

The following information was supplied regarding data availability: The raw reads are available in the NCBI Sequence Read Archive (SRA): PRJNA659620 and PRJNA659659.

## References

[ref-1] Alsaif M, Dabelah K, Featherstone R, Robinson JL (2018). Consequences of brucellosis infection during pregnancy: a systematic review of the literature. International Journal of Infectious Diseases.

[ref-2] Avila-Calderón ED, Medina-Chávez O, Flores-Romo L, Hernández-Hernández JM, Donis-Maturano L, López-Merino A, Arellano-Reynoso B, Aguilera-Arreola MG, Ruiz EA, Gomez-Lunar Z, Witonsky S, Contreras-Rodríguez A (2020). Outer membrane vesicles from *Brucella melitensis* modulate immune response and induce cytoskeleton rearrangement in peripheral blood mononuclear cells. Frontiers in Microbiology.

[ref-3] Blasco JM, Molina-Flores B (2011). Control and eradication of *Brucella melitensis* infection in sheep and goats. Veterinary Clinics of North America: Food Animal Practice.

[ref-4] Bonomi HR, Marchesini MI, Klinke S, Ugalde JE, Zylberman V, Ugalde RA, Comerci DJ, Goldbaum FA (2010). An atypical riboflavin pathway is essential for *Brucella abortus* virulence. PLOS ONE.

[ref-5] Chain PS, Comerci DJ, Tolmasky ME, Larimer FW, Malfatti SA, Vergez LM, Aguero F, Land ML, Ugalde RA, Garcia E (2005). Whole-genome analyses of speciation events in pathogenic *Brucellae*. Infection and Immunity.

[ref-6] Delpino MV, Fossati CA, Baldi PC (2009). Proinflammatory response of human osteoblastic cell lines and osteoblast-monocyte interaction upon infection with *Brucella* spp. Infection and Immunity.

[ref-7] Demars A, Lison A, Machelart A, Van Vyve M, Potemberg G, Vanderwinden J-M, De Bolle X, Letesson J-J, Muraille E (2019). Route of infection strongly impacts the host-pathogen relationship. Frontiers in Immunology.

[ref-8] Ernst J, Bar-Joseph Z (2006). STEM: a tool for the analysis of short time series gene expression data. BMC Bioinformatics.

[ref-9] Franco MP, Mulder M, Gilman RH, Smits HL (2007). Human brucellosis. Lancet Infectious Diseases.

[ref-10] Guzman-Verri C, Chaves-Olarte E, von Eichel-Streiber C, Lopez-Goni I, Thelestam M, Arvidson S, Gorvel JP, Moreno E (2001). GTPases of the Rho subfamily are required for *Brucella abortus* internalization in nonprofessional phagocytes: direct activation of Cdc42. Journal of Biological Chemistry.

[ref-11] Kotenko SV (2002). The family of IL-10-related cytokines and their receptors: related, but to what extent?. Cytokine & Growth Factor Reviews.

[ref-12] Lacey CA, Mitchell WJ, Dadelahi AS, Skyberg JA (2018). Caspase-1 and Caspase-11 mediate pyroptosis, inflammation, and control of *Brucella* joint infection. Infection and Immunity.

[ref-13] Langer JA, Cutrone EC, Kotenko S (2004). The Class II cytokine receptor (CRF2) family: overview and patterns of receptor-ligand interactions. Cytokine & Growth Factor Reviews.

[ref-14] Li Z, Wang S, Zhang H, Xi L, Zhang J, Zhang X, Zhou Q, Yi J, Li M, Zhang W, Zhang J (2018). Development and evaluation of in murine model, of an improved live-vaccine candidate against brucellosis from to *Brucella melitensis* vjbR deletion mutant. Microbial Pathogenesis.

[ref-15] Li ZQ, Shi JX, Fu WD, Zhang Y, Zhang J, Wang Z, Li TS, Chen CF, Guo F, Zhang H (2015). A *Brucella melitensis* M5-90 wboA deletion strain is attenuated and enhances vaccine efficacy. Molecular Immunology.

[ref-16] Livak KJ, Schmittgen TD (2001). Analysis of relative gene expression data using real-time quantitative PCR and the 2(-Delta Delta C(T)) method. Methods.

[ref-17] Machelart A, Van Vyve M, Potemberg G, Demars A, De Trez C, Tima HG, Vanwalleghem G, Romano M, Truyens C, Letesson J-J, Muraille E (2017). Trypanosoma infection favors *Brucella* elimination via IL-12/IFNγ-dependent pathways. Frontiers in Immunology.

[ref-18] Martín-Martín AI, Sancho P, de Miguel MJ, Fernández-Lago L, Vizcaíno N (2012). Quorum-sensing and BvrR/BvrS regulation, the type IV secretion system, cyclic glucans, and BacA in the virulence of *Brucella ovis*: similarities to and differences from smooth brucellae. Infection and Immunity.

[ref-19] Moreno E (2014). Retrospective and prospective perspectives on zoonotic brucellosis. Frontiers in Microbiology.

[ref-20] Pan G, O’Rourke K, Chinnaiyan AM, Gentz R, Ebner R, Ni J, Dixit VM (1997). The receptor for the cytotoxic ligand TRAIL. Science.

[ref-21] Pappas G, Papadimitriou P, Akritidis N, Christou L, Tsianos EV (2006). The new global map of human brucellosis. Lancet Infectious Diseases.

[ref-22] Pilson Q, Smith S, Jefferies CA, Ní Gabhann-Dromgoole J, Murphy CC (2020). miR-744-5p contributes to ocular inflammation in patients with primary Sjogrens syndrome. Scientific Reports.

[ref-23] Qureshi T, Templeton JW, Adams LG (1996). Intracellular survival of *Brucella abortus*, *Mycobacterium bovis* BCG, *Salmonella dublin*, and Salmonella *typhimurium* in macrophages from cattle genetically resistant to *Brucella abortus*. Veterinary Immunology and Immunopathology.

[ref-24] Roop RM, Barton IS, Hopersberger D, Martin DW (2021). Uncovering the hidden credentials of Brucella Virulence. Microbiology and Molecular Biology Reviews.

[ref-25] Schmittgen TD, Livak KJ (2008). Analyzing real-time PCR data by the comparative CT method. Nature Protocols.

[ref-26] Scian R, Barrionuevo P, Giambartolomei GH, De Simone EA, Vanzulli SI, Fossati CA, Baldi PC, Delpino MV (2011). Potential role of fibroblast-like synoviocytes in joint damage induced by *Brucella abortus* infection through production and induction of matrix metalloproteinases. Infection and Immunity.

[ref-27] Serer MI, Carrica MDC, Trappe J, López Romero S, Bonomi HR, Klinke S, Cerutti ML, Goldbaum FA (2019). A high-throughput screening for inhibitors of riboflavin synthase identifies novel antimicrobial compounds to treat brucellosis. FEBS Journal.

[ref-28] Soler-Llorens PF, Quance CR, Lawhon SD, Stuber TP, Edwards JF, Ficht TA, Robbe-Austerman S, O’Callaghan D, Keriel A (2016). A *Brucella* spp. Isolate from a Pac-Man Frog (Ceratophrys ornata) reveals characteristics departing from classical Brucellae. Frontiers in Cellular and Infection Microbiology.

[ref-29] Suarez-Esquivel M, Chaves-Olarte E, Moreno E, Guzman-Verri C (2020). Brucella genomics: macro and micro evolution. International Journal of Molecular Sciences.

[ref-30] Van Linthout S, Miteva K, Tschöpe C (2014). Crosstalk between fibroblasts and inflammatory cells. Cardiovascular Research.

[ref-31] Wang F, Hu S, Gao Y, Qiao Z, Liu W, Bu Z (2011a). Complete genome sequences of *Brucella melitensis* strains M28 and M5-90, with different virulence backgrounds. Journal of Bacteriology.

[ref-32] Wang F, Hu S, Liu W, Qiao Z, Gao Y, Bu Z (2011b). Deep-sequencing analysis of the mouse transcriptome response to infection with *Brucella melitensis* strains of differing virulence. PLOS ONE.

[ref-33] Wang F, Qiao Z, Hu S, Liu W, Zheng H, Liu S, Zhao X, Bu Z (2013). Comparison of genomes of *Brucella melitensis* M28 and the *B. melitensis* M5-90 derivative vaccine strain highlights the translation elongation factor Tu gene tuf2 as an attenuation-related gene. Infection and Immunity.

[ref-34] Wessells J, Baer M, Young HA, Claudio E, Brown K, Siebenlist U, Johnson PF (2004). BCL-3 and NF-kappaB p50 attenuate lipopolysaccharide-induced inflammatory responses in macrophages. Journal of Biological Chemistry.

[ref-35] Whatmore AM, Davison N, Cloeckaert A, Al Dahouk S, Zygmunt MS, Brew SD, Perrett LL, Koylass MS, Vergnaud G, Quance C, Scholz HC, Dick EJ, Hubbard G, Schlabritz-Loutsevitch NE (2014). *Brucella papionis* sp. nov., isolated from baboons (*Papio* spp.). International Journal of Systematic and Evolutionary Microbiology.

[ref-36] Xu D, Zhao J, Jiang L, Song J, Zong S, Yan X, Liu H, Zhang H, Hu S, Bu Z (2021). Comparison of transcriptional change of *B. melitensis* M5-90 after macrophage infection highlights the role of ribosome gene L31 in virulence. Veterinary Microbiology.

[ref-37] Yuan Q, Fan Y, Liu Z, Wang X, Jia M, Geng Z, Zheng J, Lu X (2020). miR-744-5p mediates lncRNA HOTTIP to regulate the proliferation and apoptosis of papillary thyroid carcinoma cells. Experimental Cell Research.

[ref-38] Zhang H, Wang B, Wu W, Deng X, Shao Z, Yi J, Wang Z, Yang N, Wang Y, Wang Y, Chen C (2020). Insights into *irr* and *rirA* gene regulation on the virulence of *Brucella melitensis* M5-90. Canadian Journal of Microbiology.

[ref-39] Zhang J, Yin S, Yi D, Zhang H, Li Z, Guo F, Chen C, Fang W, Wang J (2017). The *Brucella melitensis* M5-90ΔmanB live vaccine candidate is safer than M5-90 and confers protection against wild-type challenge in BALB/c mice. Microbial Pathogenesis.

[ref-40] Zhou D, Zhi F, Fang J, Zheng W, Li J, Zhang G, Chen L, Jin Y, Wang A (2021). RNA-Seq analysis reveals the role of Omp16 in Brucella-infected RAW264.7 cells. Frontiers in Veterinary Science.

