# Supplementary material for: Integrated mRNA-seq and miRNA-seq analysis of goat fibroblasts response to Brucella Melitensis strain M5-90
Source: PeerJ. 2021 Jun 29;9:e11679. doi: 10.7717/peerj.11679 (PMC8253117; doi:10.7717/peerj.11679)
Supplement: Supplemental Information 1 — Supplementary File for publication [file peerj-09-11679-s001.docx]

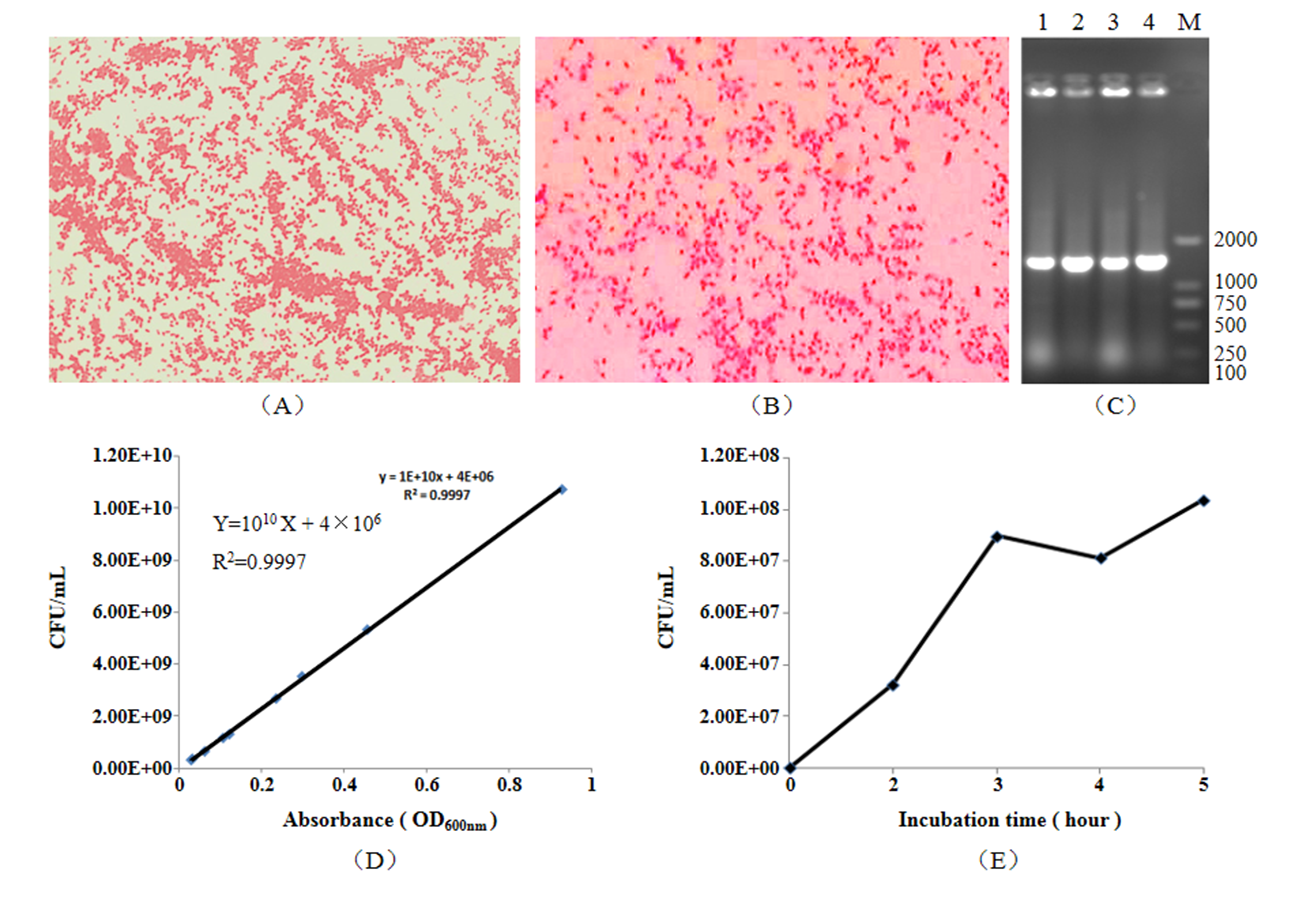


**FIGURE 1.** **Identification of *B.melitensis* M5-90 strain. (A)** Gram staining morphology of *B.melitensis* M5-90 (1000×). (**B**) Cole`s staining morphology of *B.melitensis* M5-90 (1000×). **(C)** PCR production of 16s rRNA. M, D2000 maker; lane 1-4, 16s rRNA PCR fragments. **(D)** Standard linear equation for *B.melitensis* M5-90. **(E)** Incubation time of *B.melitensis* M5-90 in goat fibroblasts (MOI=100).


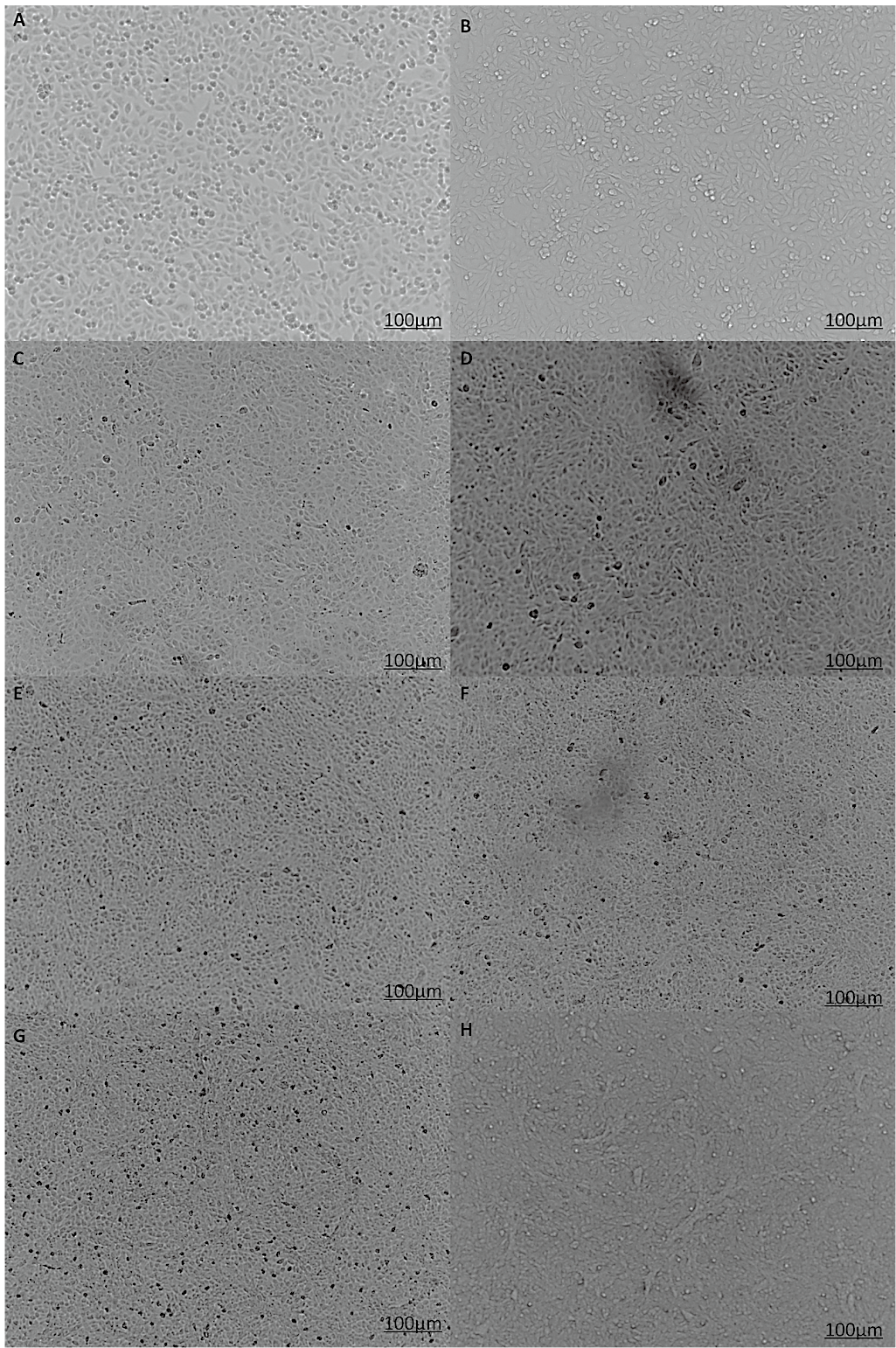


**FIGURE 2. Morphological changes of goat fibroblasts after infection with B.*melitensis* M5-90（MOI=100）**A. Goat fibroblasts not infected for 4 h; B. Goat fibroblasts infected for 4 h; C. Goat fibroblasts not infected for 24 h; D. Goat fibroblasts infected for 24 h; E. Uninfected 48 h of goat fibroblasts; F. 48 h of goat fibroblasts; G. 72 h of goat fibroblasts; H. 72 h of goat fibroblasts.


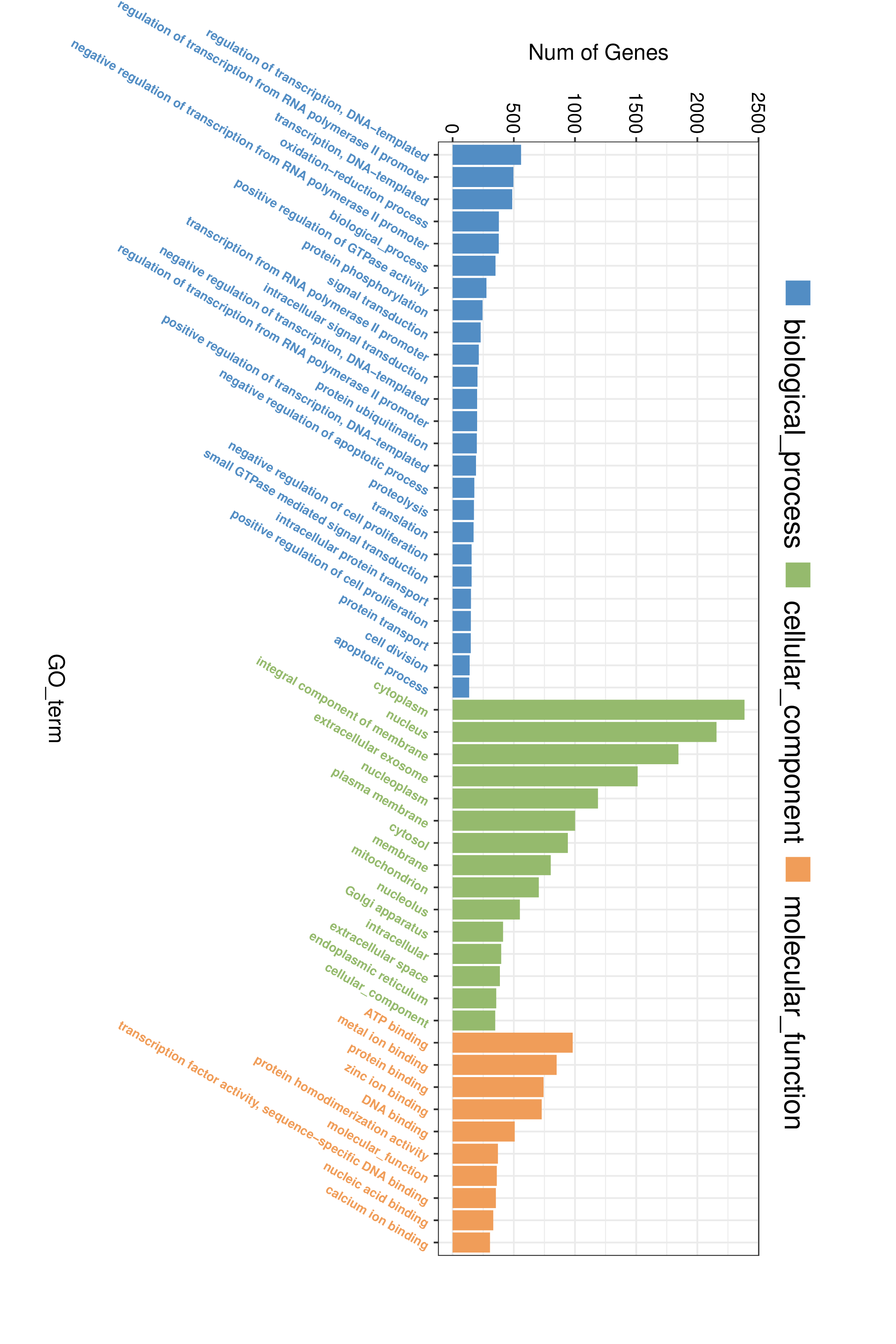


**FIGURE 3. GO enrichment analysis of all DEGs.**


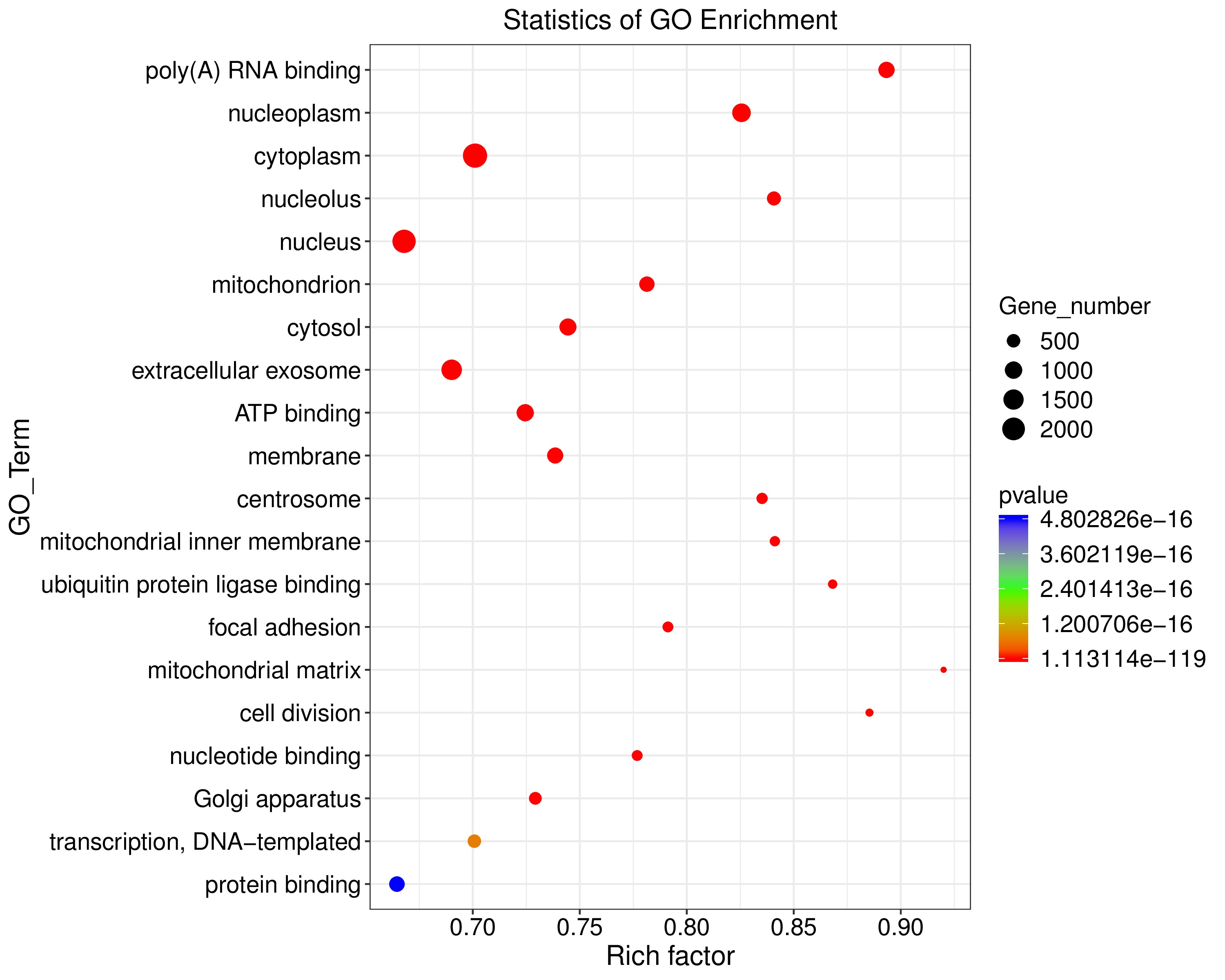


**FIGURE 4. GO enrichment scatterplot of all DEGs. (Top 20, FDR<0.05)**


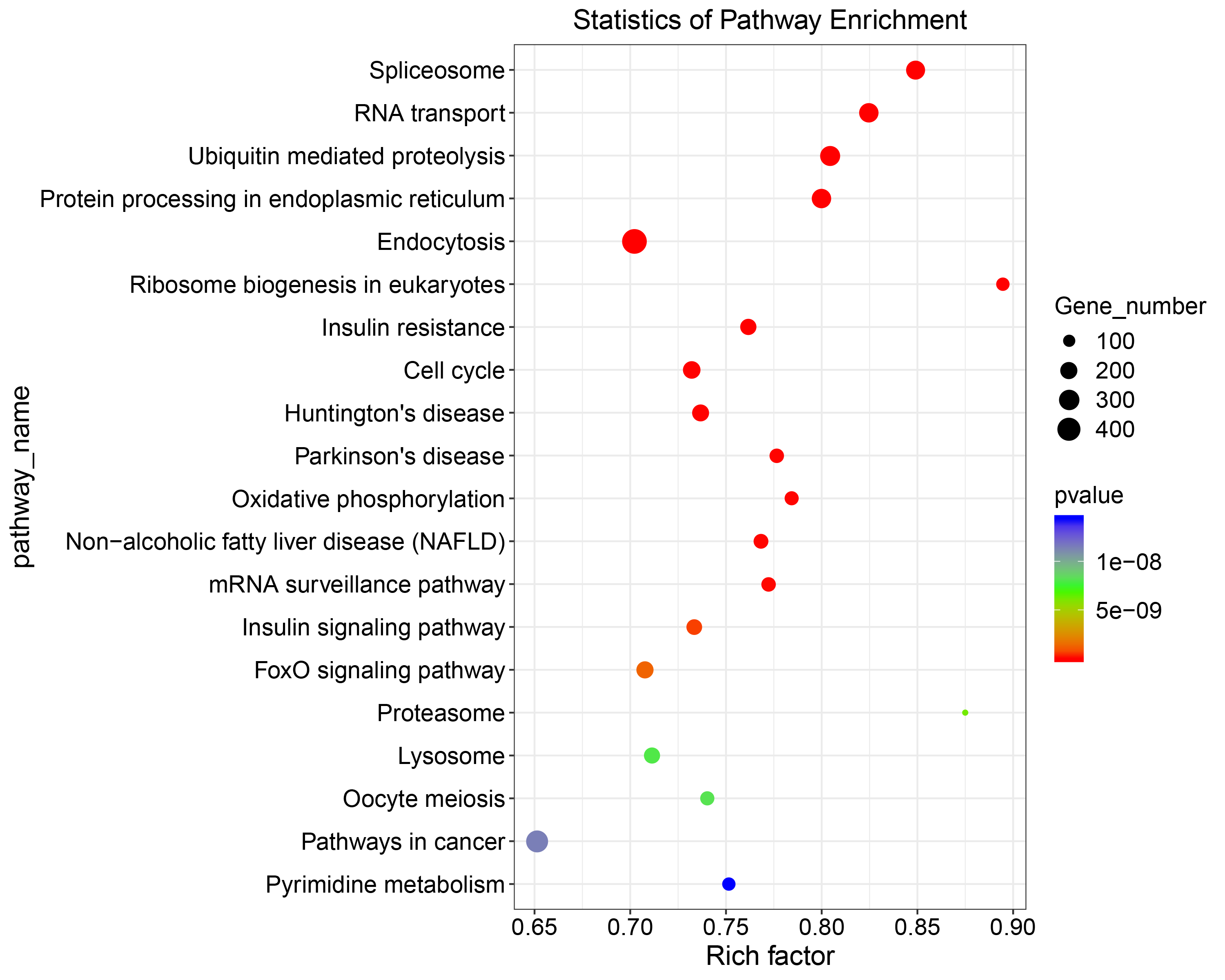


**FIGURE 5. KEGG pathway enrichment analysis of DEGs. (Top 20, FDR<0.05)**


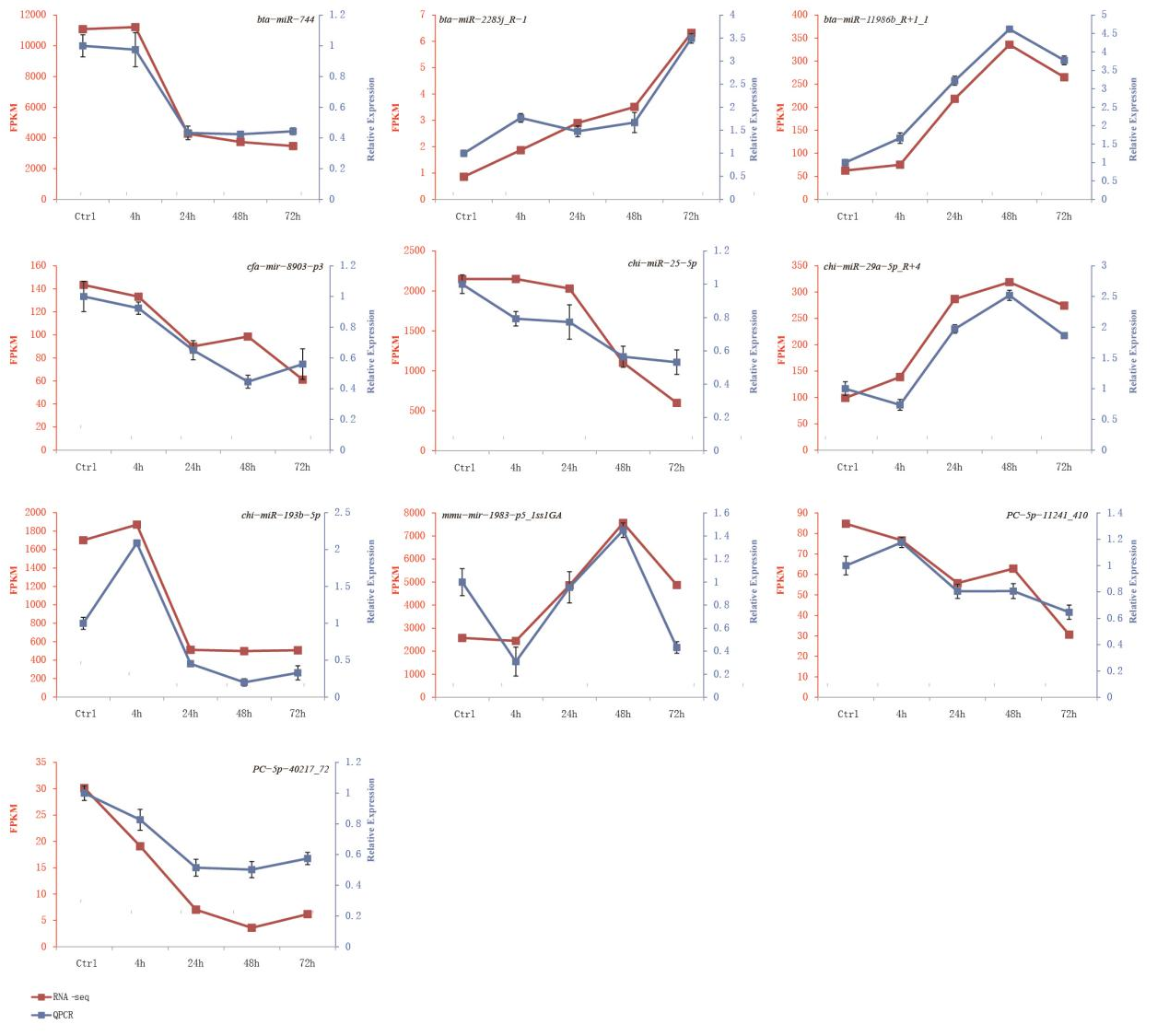


**FIGURE 6. qRT-PCR results of 10 differential expressed miRNAs.**

**Table 1 The total RNA quality**

| **Sample** | **Conc. (µg/µL)** | **OD(260/280)** | **OD(260/230)** | **Amount (µg)** | **rRNA 28S/18S** | **RIN** |
| --- | --- | --- | --- | --- | --- | --- |
| \| Ctrl-1 \| \| --- \| \| Ctrl-2 \| \| Ctrl-3 \| \| T4h-1 \| \| T4h-2 \| \| T4h-3 \| \| T24h-1 \| \| T24h-2 \| \| T24h-3 \| \| T48h-1 \| \| T48h-2 \| \| T48h-3 \| \| T72h-1 \| \| T72h-2 \| \| T72h-3 \| | \| 0.20 \| \| --- \| \| 0.20 \| \| 0.21 \| \| 0.23 \| \| 0.23 \| \| 0.23 \| \| 0.88 \| \| 0.71 \| \| 1.06 \| \| 1.37 \| \| 1.54 \| \| 1.69 \| \| 1.07 \| \| 1.22 \| \| 1.07 \| | \| 2.09 \| \| --- \| \| 2.09 \| \| 2.11 \| \| 2.05 \| \| 2.09 \| \| 2.09 \| \| 2.14 \| \| 2.13 \| \| 2.14 \| \| 2.16 \| \| 2.16 \| \| 2.17 \| \| 2.15 \| \| 2.18 \| \| 2.16 \| | \| 2.11 \| \| --- \| \| 2.05 \| \| 2.14 \| \| 1.84 \| \| 2.00 \| \| 2.11 \| \| 2.10 \| \| 2.17 \| \| 2.16 \| \| 2.14 \| \| 2.13 \| \| 2.13 \| \| 2.06 \| \| 2.05 \| \| 2.09 \| | \| 2.11 \| \| --- \| \| 2.05 \| \| 2.14 \| \| 1.84 \| \| 2.00 \| \| 2.11 \| \| 2.10 \| \| 2.17 \| \| 2.16 \| \| 2.14 \| \| 2.13 \| \| 2.13 \| \| 2.06 \| \| 2.05 \| \| 2.09 \| | \| 1.9 \| \| --- \| \| 1.9 \| \| 1.9 \| \| 1.9 \| \| 1.8 \| \| 1.9 \| \| 2.1 \| \| 2.0 \| \| 2.0 \| \| 1.9 \| \| 1.9 \| \| 1.9 \| \| 1.7 \| \| 1.9 \| \| 1.6 \| | \| 9.8 \| \| --- \| \| 9.8 \| \| 9.8 \| \| 9.7 \| \| 9.7 \| \| 9.6 \| \| 9.9 \| \| 9.8 \| \| 9.7 \| \| 9.7 \| \| 9.7 \| \| 9.7 \| \| 9.6 \| \| 9.7 \| \| 9.6 \| |

**Table 2 RNA sequencing and statistics of 15 samples used in the study**

| **Sample** | **Raw Data** | | **Valid Data** | | **Valid Ratio (reads)** | **Q20%** | **Q30%** | **GC**  **content%** |
| --- | --- | --- | --- | --- | --- | --- | --- | --- |
|  | **Read** | **Base** | **Read** | **Base** |  |  |  |  |
| Ctrl_1 | 52545332 | 7.88G | 51407804 | 7.71G | 97.84 | 99.72 | 97.32 | 49.00 |
| Ctrl_2 | 43923904 | 6.59G | 42122724 | 6.32G | 95.90 | 99.12 | 97.33 | 50.00 |
| Ctrl_3 | 52377322 | 7.86G | 51381438 | 7.71G | 98.10 | 99.48 | 94.37 | 49.00 |
| T4h_1 | 48792668 | 7.32G | 47879826 | 7.18G | 98.13 | 99.52 | 94.60 | 49.50 |
| T4h_2 | 56185414 | 8.43G | 55128232 | 8.27G | 98.12 | 99.54 | 94.81 | 49.00 |
| T4h_3 | 41997632 | 6.30G | 41173302 | 6.18G | 98.04 | 99.76 | 97.95 | 50.00 |
| T24h_1 | 42793790 | 6.42G | 41943922 | 6.29G | 98.01 | 99.78 | 98.05 | 49.50 |
| T24h_2 | 45212328 | 6.78G | 44351258 | 6.65G | 98.10 | 99.76 | 97.52 | 49.50 |
| T24h_3 | 40039252 | 6.01G | 39233130 | 5.88G | 97.99 | 99.75 | 97.60 | 49.00 |
| T48h_1 | 44037242 | 6.61G | 43168614 | 6.48G | 98.03 | 99.73 | 97.35 | 50.00 |
| T48h_2 | 54309372 | 8.15G | 53373502 | 8.01G | 98.28 | 99.52 | 94.65 | 50.00 |
| T48h_3 | 50625218 | 7.59G | 49697284 | 7.45G | 98.17 | 99.52 | 94.71 | 49.50 |
| T72h_1 | 48487368 | 7.27G | 47659452 | 7.15G | 98.29 | 99.48 | 94.46 | 50.50 |
| T72h_2 | 51944136 | 7.79G | 51005958 | 7.65G | 98.19 | 99.50 | 94.56 | 49.50 |
| T72h_3 | 49614424 | 7.44G | 48795348 | 7.32G | 98.35 | 99.43 | 94.20 | 50.50 |

**Table 3 qRT-PCR primers sequences of differentially expressed genes**

| **Gene Symbol** | **Gene Annoation** | **NCBI Reference Sequence** | **Primer Sequence (5'-3')** |
| --- | --- | --- | --- |
| *ARHGEF37* | Rho guanine nucleotide exchange factor 37 | XM_005683157.3 | F：CACTCCTGAGCAGGTATGGC  R：CTCGATGTCCTCCAGTGTCC |
| *CCL25* | C-C motif chemokine | XM_005682417.3 | F：AACTTCCAAGGCCCTCACTCT  R：CAGCCCATCTTGTGTGGGTA |
| *IFNAR2* | interferon alpha and beta receptor subunit 2 | XM_018046609.1 | F：ACGTGTGGGTAAACAGGACG  R：GGGTCCAAAGGCTTGTCTGA |
| *IL10RB* | interleukin 10 receptor subunit beta | XM_018046602.1 | F：ACCACTATCGGACCTCCCAG  R：ATGGTCCACGGTTCAGGTTC |
| *IL17RD* | interleukin 17 receptor D | XM_018038412.1 | F：GAAGCACATGATCGCTGACG  R：TTAGGAATTCGATGCCGAGGG |
| *IL18R1* | interleukin 18 receptor 1 | XM_013967547.2 | F：GACCCCTTCAGACGAGAAGC  R：GGTTCCCCTTCGATTGCAGTA |
| *IL1RN* | interleukin 1 receptor antagonist | XM_005686686.3 | F：CAAGCTCAAGTTAGAGGCTGTGA  R：AGTGATGTGCAGAGGAACCAG |
| *IL6* | interleukin 6 | NM_001285640.1 | F：ACGAGTGTGAAAACAGCAAGG  R：GCAAATCGCCTGATTGAACCC |
| *IL6ST* | interleukin 6 signal transducer | XM_005694692.3 | F：CCAGGCATTGAGGGACAAGT  R：GTAGCCACCTCGTCTCACAG |
| *IL7R* | interleukin 7 receptor | XM_005694804.3 | F：CAGTCAGCTGGAAGTGGATGG  R：CAAAAGCGCCCCACATATTTCA |
| *INHBB* | inhibin beta B subunit | XM_018061661.1 | F：ACTCCTAGCACTTGCACACC  R：ACATGGCACTTGGACCTCTG |
| *NFKB1* | nuclear factor kappa B subunit 1 | XM_018049264.1 | F：CCCACTATGGATTCCCCACG  R：TCTGTCGTCACTCTTGCCAC |
| *RELT* | RELT tumor necrosis factor receptor | XM_018059371.1 | F：TGTAACCTGCTCAAGCGGAA  R：GCGGCATTCTCTTTCTTCTCTG |
| *SOS2* | SOS Ras/Rho guanine nucleotide exchange factor 2 | XM_018054371.1 | F：TGAGGAACGGGTGGCAATAC  R：TTCTTTCCTGCAACGCCTCA |
| *STAT3* | signal transducer and activator of transcription 3 | XM_013972399.2 | F：ACCTGGAGACGCACTCATTG  R：CACTTGATCCCACGTTCCGA |
| *TGFB3* | transforming growth factor beta 3 | XM_005686141.3 | F：ATTGCTTCCGCAATCTGGAGG  R：GTGCTGTGGGTTGTGTCTGA |
| *TGFBR2* | transforming growth factor beta receptor 2 | XM_018067217.1 | F：CTGCTTGGGTTTTGGAAGCC  R：ATGAGCAACAGCGATTGGGA |
| *TNFRSF21* | TNF receptor superfamily member 21 | XM_018039101.1 | F：TGGCCACGGTATTGACATCC  R：TGTACCCGTTGGAGAAAGCG |
| *TNFSF15* | tumor necrosis factor superfamily member 15 | XM_005684350.3 | F：CACCTGACAGTTGTGAGACAGA  R：TATGGTCCCTCGGAAGGTCA |
| *VAV2* | vav guanine nucleotide exchange factor 2 | XM_018056166.1 | F：GGACTTCGGGAAGGTCATCTC  R：CCTCGTCATTCTCCGTGGTC |

**Table 4 Specific stem-loop and forward primers of DE miRNAs**

| **miRNAs** | **Stem-loop Primer**  **Sequence (5'-3')** | **Forward Primer**  **Sequence (5'-3')** |
| --- | --- | --- |
| *chi-miR-10b-3p* | GTCGTATCCAGTGCAGGGTCCGAGGTATTCGCACTGGATACGACATTCCCCT | CGCACAGATTCGATTCTAGG |
| *bta-miR-744* | GTCGTATCCAGTGCAGGGTCCGAGGTATTCGCACTGGATACGACTGCTGTTA | TGCGGGGCTAGGGCTA |
| *PC-5p-40217_72* | GTCGTATCCAGTGCAGGGTCCGAGGTATTCGCACTGGATACGACAGAGGGCA | GCGCGAAGGTGACTTTTTAT |
| *PC-5p-11241_410* | GTCGTATCCAGTGCAGGGTCCGAGGTATTCGCACTGGATACGACGCCCCCGGA | ATATAGCGGGCGAGGGTCC |
| *chi-miR-25-5p* | GTCGTATCCAGTGCAGGGTCCGAGGTATTCGCACTGGATACGACAGCAATTG | AGGCGGAGACTTGGGC |
| *bta-miR-11986b_R+1_1* | GTCGTATCCAGTGCAGGGTCCGAGGTATTCGCACTGGATACGACTAAAGGAC | GCGCTTTTCCAATGAGTTAGT |
| *mmu-miR-1983-P5_1ss1GA* | GTCGTATCCAGTGCAGGGTCCGAGGTATTCGCACTGGATACGACGCGCCACT | AAAAGCATGCTCCAGTGGC |
| *chi-miR-29a-5p_R+4* | GTCGTATCCAGTGCAGGGTCCGAGGTATTCGCACTGGATACGACACTCTGAA | GCGACTGATTTCTTTTGGTGT |
| *bta-miR-2285j_R-1* | GTCGTATCCAGTGCAGGGTCCGAGGTATTCGCACTGGATACGACAAAAAGTT | CGCAAAAACCAGAACGAAC |
| *cfa-mir-8903-p3* | GTCGTATCCAGTGCAGGGTCCGAGGTATTCGCACTGGATACGACATTCCCAG | ATCTGGGGTAGGGCCTG |
| *chi-miR-193b-5p* | GTCGTATCCAGTGCAGGGTCCGAGGTATTCGCACTGGATACGACTCATCTCG | CGGGGTTTTGAGGGC |

**Additional information:**

**mRNA Sequencing and Data Analysis**

Cutadapt software (https://cutadapt.readthedocs.io/en/stable/,version:cutadapt-1.9) was used to remove the reads that contained adaptor contamination, (command line:~cutadapt -a ADAPT1 -A ADAPT2 -o out1.fastq -p out2.fastq in1.fastq in2.fastq -O 5 -m 100). After removed the low quality bases and undetermined bases, we used HISAT2 software (https://daehwankimlab.github.io/hisat2/, version:hisat2-2.0.4) to map reads to the genome (ftp://ftp.ncbi.nlm.nih.gov/genomes/all/GCF/001/704/415/GCF_001704415.1_ARS1/GCF_001704415.1_ARS1_genomic.fna.gz), (command line:~hisat2 -1 R1.fastq.gz -2 R1.fastq.gz -S sample_mapped.sam). The mapped reads of each sample were assembled using StringTie (http://ccb.jhu.edu/software/stringtie/,version:stringtie-1.3.4d.Linux_x86_64) with default parameters (command line:~stringtie -p 4 -G genome.gtf -o output.gtf -I sample input.bam). Then, all transcriptomes from all samples were merged to reconstruct a comprehensive transcriptome using gffcompare software (http://ccb.jhu.edu./software/stringtie/gffcompare.shtml,versioin:gffcompare-0.9.8. Linux_x86_64). After the final transcriptome was generated, StringTie and ballgown (http://www.bioconductor.org/packages/release/bioc/html/ballgown.html) were used to estimate the expression levels of all transcripts and perform expression level for mRNAs by calculating FPKM (FPKM = [total_exon_fragments / mapped_reads(millions) × exon_length(kB)]),(command line:~stringtie -e -B -p 4 -G merged.gtf -o samples.gtf samples.bam). The differentially expressed mRNAs were selected with fold change > 2 or fold change < 0.5 and p< 0.05 by R package edgeR (https://bioconductor.org/packages/release/biocI/html/edgeR.html) and then analysis GO enrichment and KEGG enrichment to the differentially expressed mRNAs.

**miRNA Sequencing and Data Analysis**

Raw reads were subjected to an in-house program, ACGT101-miR (LC Sciences, Houston, Texas, USA) to remove adapter dimers, junk, low complexity, common RNA families (rRNA, tRNA, snRNA, snoRNA) and repeats. Subsequently, unique sequences with length in 18~26 nucleotide were mapped to specific species precursors in miRBase 22.0 by BLAST search to identify known miRNAs and novel 3p- and 5p- derived miRNAs. Length variation at both 3’ and 5’ ends and one mismatch inside of the sequence were allowed in the alignment. The unique sequences mapping to specific species mature miRNAs in hairpin arms were identified as known miRNAs. The unique sequences mapping to the other arm of known specific species precursor hairpin opposite to the annotated mature miRNA-containing arm were considered to be novel 5p- or 3p derived miRNA candidates. The remaining sequences were mapped to other selected species precursors (with the exclusion of specific species) in miRBase 22.0 by BLAST search, and the mapped pre-miRNAs were further BLASTed against the specific species genomes to determine their genomic locations. The above two we defined as known miRNAs. The unmapped sequences were BLASTed against the specific genomes, and the hairpin RNA structures containing sequences were predicated from the flank 80 nt sequences using RNAfold software (http://rna.tbi.univie.ac. at/cgi-bin/RNAfold.cgi). The criteria for secondary structure prediction were:(1) number of nucleotides in one bulge in stem (≤12) (2) number of base pairs in the stem region of the predicted hairpin (≥16) (3) cutoff of free energy (kCal/mol ≤-15) (4) length of hairpin (up and down stems +terminal loop ≥50) (5) length of hairpin loop (≤20). (6) number of nucleotides in one bulge in mature region (≤8) (7) number of biased errors in one bulge in mature region (≤4) (8) number of biased bulges in mature region (≤2) (9) number of errors in mature region (≤7) (10) number of base pairs in the mature region of the predicted hairpin (≥12) (11) percent of mature in stem (≥80). Differential expression of miRNAs based on normalized deep-sequencing counts were analyzed by ANOVA. The significance threshold was set to be 0.05. To predict the genes targeted by most abundant miRNAs, two computational target prediction algorithms (TargetScan and Miranda) were used to identify miRNA binding sites. Finally, the data predicted by both algorithms were combined and the overlaps were calculated. The GO terms and KEGG Pathway of these most abundant miRNAs, miRNA targets were also annotated.
